# Structural–functional dissection and characterization of yield-contributing traits originating from a group 7 chromosome of the wheatgrass species *Thinopyrum ponticum* after transfer into durum wheat

**DOI:** 10.1093/jxb/ert393

**Published:** 2013-12-06

**Authors:** Ljiljana Kuzmanović, Andrea Gennaro, Stefano Benedettelli, Ian C. Dodd, Stephen A. Quarrie, Carla Ceoloni

**Affiliations:** ^1^Department of Agriculture, Forestry, Nature and Energy (DAFNE), University of Tuscia, Viterbo, Italy; ^2^Department of Plant, Soil and Environmental Sciences, University of Florence, Florence, Italy; ^3^The Lancaster Environment Centre, Lancaster University, Lancaster, UK; ^4^Faculty of Biology, Belgrade University, Belgrade, Serbia

**Keywords:** Alien gene transfer, biomass, chromosome engineering, flag leaf, grain yield, seed number, segregation distortion, tiller number, *Triticum aestivum*, *Triticum durum*, yield QTL.

## Abstract

For the first time, using chromosome engineering of durum wheat, the underlying genetic determinants of a yield-improving segment from *Thinopyrum ponticum* (7AgL) were dissected. Three durum wheat–*Th. ponticum* near-isogenic recombinant lines (NIRLs), with distal portions of their 7AL arm (fractional lengths 0.77, 0.72, and 0.60) replaced by alien chromatin, were field-tested for two seasons under rainfed conditions. Yield traits and other agronomic characteristics of the main shoot and whole plant were measured. Loci for seed number per ear and per spikelet were detected in the proximal 7AgL segment (0.60–0.72). Loci determining considerable increases of flag leaf width and area, productive tiller number per plant, biomass per plant, and grain yield per plant were located in the distally adjacent 0.72–0.77 7AgL segment, while in the most distal portion (0.77–1.00) genetic effects on spikelet number per ear were identified. Contrary to previous reports, trials with the bread wheat T4 translocation line, carrying on 7DL a sizeable 7AgL segment of which those present in the durum wheat-*Th. ponticum* NIRLs represent fractions, gave no yield advantage. The hypothesis that ABA might be a factor contributing to the 7AgL effects was tested by analysing endogenous ABA contents of the NIRLs and their responses to exogenous ABA application. The 7AgL yield-related loci were shown to be ABA-independent. This study highlights the value of wheat–alien recombinant lines for dissecting the genetic and physiological basis of complex traits present in wild germplasm, and provides a basis for their targeted exploitation in wheat breeding.

## Introduction

Wheat is the most cultivated cereal worldwide, feeding about 40% of the human population (FAO, 2009). Furthermore, in the light of global climatic changes, the better adaptability of wheat to semi-arid conditions than maize and rice, the other two major staple crops, is expected to favour its expanded cultivation on a world scale (Dixon *et al.*, 2009). After the rapid yield increases of the Green Revolution in developing countries during 1966–1979 of around 3.6% per year, annual yield increases during 1995–2005 have declined to only 1.1% (Dixon *et al.*, 2009). This current genetic ‘bottleneck’ coupled with the increased challenges for wheat production of the current millennium make it difficult to meet the required yield increments without the application of unconventional breeding strategies (targeted genome manipulations). To substantiate the efforts of a ‘knowledge-intensive’ 21st century Green Revolution (Evans, 2009), the new genotyping and phenotyping technologies (Tester and Langridge, 2010) represent essential tools effectively to survey, reliably characterize, and hence exploit the genetic diversity present in the crop germplasm pools, including those of the wild crop relatives (Gennaro *et al.*, 2007; Mba *et al.*, 2012; Hawkesford *et al.*, 2013; King *et al.*, 2013). Moreover, thanks to the extensive synteny among the many species of the Triticeae tribe and whole Poaceae family (Sorrells *et al.*, 2003; Devos, 2010), genomic mapping and sequence information is highly transferable across species.

Combining molecular and phenotypic characterization of the available natural variation can be particularly valuable for genetic dissection of complex traits such as yield, typically having a multigenic control by several quantitative trait loci (QTL), a relatively low heritability and a significant interaction with environmental conditions. The development of molecular marker platforms and dense genetic maps of wheat allowed numerous QTL linkage analyses to be carried out on yield and associated traits, revealing the involvement of many loci around the genome (Quarrie *et al.*, 2005; Marza *et al.*, 2006; Snape *et al.*, 2007; Maccaferri *et al.*, 2008; McIntyre *et al.*, 2010; Tsilo *et al.*, 2010).

The search for loci underlying yield-related traits extends to ‘non-crop’ species, including wild relatives, land races, and other non-adapted genetic materials, which display a wealth of potentially useful features for crop improvement, along with undesirable ones. Indeed, the ability to transfer only the defined, target alien loci and eliminate unwanted ones is the key to harnessing alien genetic variation, making it an effective way to counter problems of crop genetic erosion (Tester and Langridge, 2010). Cultivated wheats, particularly the hexaploid bread wheat (*Triticum aestivum* L., 2*n*=6*x*=42, genome AABBDD), but also the tetraploid durum wheat (*T. durum* L. var. *durum*, 2*n*=4*x*=28, genome AABB), are amenable to reach this goal through ‘chromosome engineering’ approaches (Ceoloni and Jauhar, 2006; Qi *et al.*, 2007). They can in fact benefit from the unique richness of cytogenetic materials that have greatly facilitated knowledge and controlled manipulation of their genomes and those of related Triticeae species. Since the mid-20th century, chromosome engineering methodologies have enabled access to chromosomes of even distant relatives, sharing only partial homology, i.e. homoeology, with those of wheat, and the transfer of several genes, mainly controlling resistance to various diseases, but also abiotic stress tolerance and quality traits (Friebe *et al.*, 1996; Ceoloni and Jauhar, 2006; Colmer *et al.*, 2006).

Among several member species of the vast wheatgrass genus *Thinopyrum* Löve, the decaploid *Thinopyrum ponticum* (2*n*=10*x*=70; syn. *Agropyron elongatum*, *Lophopyrum ponticum, Elytrigia pontica*) is one of the most extensively exploited (Ceoloni *et al.*, 2014). Several major genes and QTL of proved or potential breeding value are concentrated on the long arm of a chromosome homoeologous to those of wheat group 7, originally named 7Ag (Sears, 1973) or 7el (Sharma and Knott, 1966). When introduced into wheat cultivars in the form of substitution and translocation lines, 7Ag chromosomes of different *Th. ponticum* accessions revealed the presence of genes controlling resistance to several wheat diseases, including rusts (e.g. *Lr19*, *Sr25*) and scab, as well as genes affecting grain pigment content (*Yp*) and even yield (see Ceoloni *et al.*, 2014, and references therein).

The existence of loci positively affecting yield in wheat–*Th. ponticum* genetic stocks was originally suggested on the basis of results obtained by CIMMYT (Singh *et al.*, 1998; Reynolds *et al.*, 1999, 2001, 2005; Monneveux *et al.*, 2003), using near-isogenic lines (NILs) of the original T4 (=Agatha) translocation (70% of the 7AgL arm onto the wheat arm 7DL; Sharma and Knott, 1966). In all bread wheat backgrounds studied, the 7AgL translocation was associated with increased yield, biomass and grain number per ear. Preliminary evidence suggested the higher number of grains per ear to result from reduced floral abortion which was related to lower abscisic acid (ABA) concentrations in spike tissue at the boot stage (Reynolds *et al.*, 2008). Notwithstanding this, the mechanism of ABA effect on spike fertility remains poorly understood. High ABA concentrations in the wheat spike, and floral organs in particular, are associated with lower spike fertility, especially under water-stress conditions (Morgan, 1980; Morgan and King, 1984; Zeng and King, 1986; Westgate *et al.*, 1996), and the most productive and fertile wheat genotypes maintain the lowest ABA level around pollen meiosis (Cao *et al.*, 2000), a key stage determining wheat spike fertility. Positive effects of the 7AgL translocation were particularly evident in the absence of water deficit (Singh *et al.*, 1998), although 7AgL effects varied with genetic background and were environment- independent (Sibikeev *et al.*, 2000; Monneveux *et al.*, 2003).

Distal portions of the same 7AgL segment present in line T4 were separately introduced into the 7AL arm of durum wheat recombinant lines, with the primary aim of transferring the *Lr19+Yp+Sr25* linked genes (Gennaro *et al.*, 2003; Ceoloni *et al.*, 2005). During multi-location field tests conducted for several years in Central Italy, derivatives of recombinant line R5-2–10, including all target genes in a 23% long 7AgL terminal segment, not only yielded significantly more than varietal controls following heavy leaf-rust epidemics, but also demonstrated their intrinsically good yield potential in virtually rust-free seasons. Though not consistent across years and environments, significant increments in number of grains per ear or in thousand-grain weight were observed (Gennaro *et al.*, 2003, 2007).

Line R5-2–10 is one of several durum wheat-*Th. ponticum* recombinant lines produced by Ceoloni *et al.* (2005) through chromosome engineering. Their recombinant chromosomes define a series of physical breakpoints concentrated in the distal half of the 7AL-7AgL arms, with the maximum size of a distal 7AgL segment spanning around 40% of the arm. In the largely syntenic and colinear 7AL region (Ceoloni *et al.*, 2014), several loci for yield-contributing traits in both bread and durum wheat have been reported. In bread wheat, a QTL stably expressed across environments, affecting mainly grain number per ear, was identified on distal 7AL (Quarrie *et al.*, 2005). Its expression was associated with a gene(s) controlling flag leaf width and chlorophyll content, indirectly affecting the amount of assimilates transferred to the spike (Quarrie *et al.*, 2006). Furthermore, this QTL was detected following an exogenous ABA treatment in controlled environments (Quarrie *et al.*, 2007). Loci at similar positions on 7AL have also been found for traits such as grain yield, grain number per ear or per unit area, and spikes per plant (Kumar *et al.*, 2007; McIntyre *et al.*, 2010; Wu *et al.*, 2012), as well as kernel weight and size (Marza *et al.*, 2006; Tsilo *et al.*, 2010; Bennett *et al.*, 2012; Wang *et al.*, 2012*a*). Similarly, in durum wheat, multiple loci for traits such as thousand-kernel weight and grain number per unit area were particularly concentrated toward the distal end of 7AL (Maccaferri *et al.*, 2011).

Given the substantial colinearity evident from comparing group 7 wheat versus *Th. ponticum* genetic maps (Ceoloni *et al.*, 2014), and the reasonable hypothesis of the existence of multiple yield-contributing loci along the 7L arms of both species, the durum wheat recombinants carrying different amounts of 7AgL chromatin on their 7AL should be suitable tools to explore different physical regions of the homoeologous arms and to characterize effects of their possible homoeo-loci for yield and yield-related traits. A similar approach enabled detection and analysis of genetic components of root traits present on the short arm of rye chromosome 1 (1RS) introduced into bread wheat (Sharma *et al.*, 2011). Here, for the first time, the effects in durum wheat of several yield-contributing traits of wild (*Th. ponticum*) origin was examined and, thanks to the use of near-isogenic recombinant lines (NIRLs) with 7AgL segments of varying lengths, assigned to defined 7AgL sections their responsible loci As a result, recombinant genotypes with a combination of novel alien attributes, including yield-enhancing traits, and great potential for breeding exploitation have been identified. The hypothesis that ABA is a factor contributing to the 7AgL effects was also tested, by measuring its endogenous content in immature spikes, and analysing the effects of ABA application at the double ridge stage of development.

## Materials and methods

### Plant material

Derivatives of three durum wheat–*Th. ponticum* recombinant lines from the original set developed by Ceoloni *et al.* (2005) were used, named R23-1, R112-4, and R5-2–10. Each line is characterized by the replacement of a distal segment of its 7A long arm (7AL) by homoeologous 7AgL *Th. ponticum* chromatin, including the *Lr19+Yp+Sr25* genes in the shared distal portion, but with a varying position of the proximal breakpoint (BP), hence spanning from 23% to 40% of the arm (Ceoloni *et al.*, 2005, 2014). Within the physical intervals defined by the different BPs, several molecular markers of various types were located (Kuzmanović, 2011; Ceoloni *et al.*, 2014). In the present study, NIRLs were used for all the three genotypes, with cv. Simeto contributing the prevailing background during backcrossing (BC). In particular, homozygous carriers (HOM+) and non-carriers (HOM–) of the corresponding 7AgL segment were represented by BC_5_F_4–5_ plants for R5-2–10 and R112-4, and BC_4_F_4–5_ plants for R23-1. The NIL of the bread wheat T4 translocation line in the background of cv. Thatcher (here referred to as T4) was also included in the experiment together with cv. Thatcher.

### Growth conditions

During the two experimental years (2009 and 2010), glasshouse and field locations were at the experimental station of the University of Tuscia, Viterbo, Central Italy (Lat: 42.426418° N Long: 12.080573° E). Seeds were surface-sterilized and pre-germinated for 2–3 d at 22 °C. Once seminal roots developed, plants were transferred into 6×6×6cm pots with BRILL Type 3 special substrate, and placed in a growth chamber at 5 °C and a 12h photoperiod for 3 weeks vernalization. They were then transplanted into 1.8 l pots (diameter 16cm; depth 13cm) of the same substrate supplemented with 1g l^–1^ of Triabon^®^, a slow-release fertilizer (N16-P8-K12-Mg4 plus trace elements), and after a 2-week period at around 12/16 °C (night/day), transferred to a glasshouse for ABA treatment (around mid-March; see Supplementary Fig. S1 at *JXB* online). Temperature and light conditions were not controlled, though diurnal variations remained relatively stable throughout the treatment period (see Supplementary Fig. S1 at *JXB* online). While in the glasshouse, plants were regularly watered with the same amount of water per pot and protected against fungal pathogen attack at the 2–3 leaf stage by mild fungicide treatment with 4ml l^–1^ Folicur^®^. Following ABA treatment, treated and control plants were kept in the glasshouse for about two more weeks and then transferred to the field, remaining until harvest in the same pots inserted within the soil. Plants were organized into three randomized replicated blocks, arranged in four pots per row (row length of 80cm). Once in the field, plants were grown under rainfed conditions, and appropriate weed, disease, and pest control measures were applied. The growing season spanned from 12 February until mid-June in 2009, and from 22 January until the first 10 d of July in 2010. Meteorological parameters of the two experimental years are illustrated in Supplementary Fig. S1 at *JXB* online. Compared with 2010, the 2009 growing season was characterized by a considerably warmer spring, accompanied by less rainfall, particularly around flowering time. Remarkably, at heading time, typically in May, maximum temperatures ranged from 18 °C to 32 °C in 2009, while from 13 °C to 26 °C in 2010; in the same period, rainfall amounted to 11.6mm and 185mm, respectively.

### Treatment with exogenous ABA

ABA treatment of the main shoot was performed when the plants had reached the ‘double ridge’ stage (Zadoks 20; Zadoks *et al.*, 1974), when initiation of the main shoot leaves is finished and the ear floral structure (spikelets and florets) initiates (Rawson and Macpherson, 2000). This allowed ABA to affect leaf development of the treated shoot, and spike productivity traits such as number of seeds and grain yield. The double ridge stage was determined by dissecting spare plants grown in the same experimental conditions (see Supplementary Fig. S2 at *JXB* online). Half the plants of each genotype were then treated with 3.8×10^–4^ M ABA solution containing 0.1% TWEEN^®^ (Sigma), and the other half with 0.1% TWEEN^®^, used as a control. ABA solution or 0.1% TWEEN^®^ (25μl) was placed on each of two basal leaves just next to the leaf sheath (see Supplementary Fig. S3 at *JXB* online). A hole was then carefully made with a needle (diameter 0.45mm) through the drop and leaf blade to ensure immediate uptake of the solution by the plant. The same treatment was repeated 3 d later.

### Measuring yield and yield-related traits

Toward the end of grain-filling period (Zadoks 77), while the leaves were still green and hydrated, width (FLW, FL-1W) and length (FLL, FL-1L) of the flag leaf and flag leaf-1 of the main shoot were measured, and the area of the two leaves (FLA, FL-1A) calculated as their respective length×width×0.75 conversion factor (Dodig *et al.*, 2010). At maturity and at post-harvest, total height (TH), ear length (EL1), seed number per ear (SNE1), spikelet number per ear (SPNE1), seeds per spikelet (SPS1), and grain yield per ear (GYE1) of the main shoot were recorded. Number of days to heading (DTH) was recorded when the ear was half out of the flag leaf sheath; at heading time tiller number per plant (TTH) was also recorded. Traits of the whole plant measured at maturity were: total productive tiller number (TTN), grain yield per plant (GY) and per ear (GYE), seed number per plant (SN) and per ear (SNE), and thousand-kernel weight (TKW). In 2010, dry biomass (B) of the whole above-ground part of the plant was also weighed and harvest index (HI) calculated.

For traits recorded on the main shoot, 40–100 HOM+ and HOM– plants/NIRL, deriving from two sister self progeny/NIRL, were used over the two years. Fewer individuals were used for whole-plant measurements, consisting of 30–70 HOM+ and HOM– plants/NIRL over the two years.

### Assessing juvenile ear ABA content and leaf relative water content (RWC)

Plants were grown in the field in 2010 as described above. They were arranged to give rows of 6 plants, at 20cm spacing between plants and between rows, each row corresponding to one of two sister lines of each genotype (HOM+ or HOM–, see above). Plants were regularly watered throughout the growth period, and at the ‘boot stage’ (Zadoks 45; see Supplementary Fig. S4 at *JXB* online) ears of the main shoots were detached, frozen immediately in liquid nitrogen, and stored at –80 °C until use. Immature spikes were then lyophilized in a freeze-dryer at –50 °C for 48h, then immediately homogenized to a powder, by a planetary ball mill (Retsch^®^, PM100).

ABA was extracted from about 0.2g of each pulverized ear sample by adding de-ionized water in a 1:30 ratio and shaking overnight at 5 °C. The supernatant was directly used for ABA radioimmunoassay (RIA) according to Quarrie *et al.* (1988). As aqueous extracts of barley, maize, and wheat tissues were reported to have less than 10% contamination (Quarrie *et al.*, 1988), ABA concentrations recorded in this study, expressed on a dry weight basis, have not been corrected for contamination.

At the same time as sampling the juvenile ear, the flag leaf of the same plant was sampled (four discs of 1cm diameter) to measure its RWC, which was calculated with the formula RWC=(FW–DW)/(TW–-DW); FW, fresh weight at the moment of sampling; TW, turgid weight after 2h floating on distilled water in the dark at room temperature; DW: dry weight after overnight drying at 65 °C.

### Statistical analysis

To investigate the effects of genetic and/or environmental factors and interactions between them on recorded variables, an analysis of variance (ANOVA) was conducted applying to the main shoot and whole plant datasets a general linear model (ANOVA-GLM) as a mixed effect model, using SYSTAT12 software (Systat Software Incorporated, San Jose, CA, USA). Each variable (i.e. trait measured) was entered as a ‘dependent’ factor against ‘independent’ factors. The latter were genotype background (G), i.e. background genetic information from the recurrent variety, year (Y), and treatment with ABA (T), as well as presence/absence of the 7AgL segment nested in the background [7AgL(G)], and year-nested replica [R(Y)] used in the model as the error. Year was considered a random effect factor because the experiments were replicated over two years using the same experimental conditions by using the same design. First order [Y×G, Y×T, 7AgL(G), and R(Y)], second order [Y×7AgL(G), T×7AgL(G)], and third order [Y×T×7AgL(G)] interactions between the above factors were analysed as well. Three levels of significance were considered, corresponding to *P* <0.05, *P* <0.01 and *P* <0.001. When significant interactions (*F* values) were observed, a pairwise analysis was carried out by the Tukey Honestly-Significant-Difference (HSD) at the 0.95 confidence level.

Data for durum wheat yield and yield-related traits recorded on the reduced subset of individuals (see above) were also subjected to discriminant analysis (DA) using SYSTAT12, excluding the traits that were derivatives of other traits (i.e. TKW, SPS, HI). Two sets of data were subjected to DA, i.e. those corresponding to traits measured in both years (2009 and 2010), and those concerning year 2010 only, when the total biomass trait was also evaluated. The classification criterion adopted in DA was the combination of ‘genotype’ and ‘7AgL segment’ presence/absence. The procedure applied for verification of discriminant power of each variable was ‘Complete’ with 0.001 tolerance. Data were plotted on the basis of the first two scores obtained from DA with NTSYSpc 2.20N software package (Rohlf, 2008).

ANOVA–GLM was also applied to analyse endogenous ABA contents in juvenile ears and corresponding RWCs, with G and 7AgL(G) included as independent factors, followed by the Tukey HSD test at the 0.95 confidence level when significant effects of factor or interaction were observed.

## Results

### Yield and yield-related traits of durum wheat NIRLs

To investigate all sources of variation for yield and yield-related traits, the complete data sets (both whole plant and main shoot traits) for the durum wheat genotypes grown over two consecutive years (HOM+ and HOM– individuals for each NIRL, both ABA- and water-treated on the main shoot) were subjected to ANOVA–GLM (Table 1). The models explained 18–83% of the observed variability (*R* ^2^) for the whole plant traits and 16–67% for the main shoot traits analysed, and showed different levels of significance of the individual independent factors or of their interactions. Overall (whole plant and main shoot traits), the trait variability was low to medium, as shown by the observed coefficients of variation (CV; see Supplementary Table S1 at *JXB* online).

**Table 1.** Mean squares from ANOVA-GLM for yield-related traits of the three homozygous durum wheat NIRLs (HOM+) and of their 7AgL non-carrier controls (HOM−) determined for whole plant in (A) 2009 and 2010, (B) 2010 only, and (C) for the main shoot in 2009 and 2010 GY, grain yield per plant (g); SN, seed number per plant; TTN, total productive tiller number; TTH, tiller number per plant at heading; TKW, thousand-kernel weight (g); GYE, grain yield per ear (g); SNE, seed number per ear; TH, plant height (mm); DTH, days to heading; B, biomass per plant (g); HI, harvest index; FLW, flag leaf width (cm); FLL, flag leaf length (cm); FLA, flag leaf area (cm^2^); FL-1W, flag leaf-1 width (cm); FL-1L, flag leaf-1 length (cm); FL-1A, flag leaf-1 area (cm^2^); EL1, ear length (cm); SNE1, seed number per main shoot ear; SPNE1, spikelet number per main shoot ear; SPS1, seeds per spikelet; GYE1, grain yield per main shoot ear (g); *, **, *** indicate significant *F* values at *P* <0.05, 0.01, and 0.001, respectively.

**Factor**	**Df**	**(A)**										
		**GY**	**SN**	**TTN**	**TTH**	**TKW**	**GYE**	**SNE**	**TH**	**DTH**		
G	2	1566.31***	77676.42*	39.79*	94.09**	369.00***	4.54***	11.83	134308.35***	1085.63***		
Y	1	267.83*	784953.58***	49.45*	19.39	2877.28***	3.64***	6041.08***	23221.88*	3835.30***		
T	1	10.05	12675.15	1.22	1.80	3.13	0.10	1.98	5239.95	1.12		
YxG	2	912.88***	104914.10*	8.99	64.42**	22.74	5.25***	698.77***	78085.87***	1.93		
YxT	1	6.24	3237.38	35.03	51.31	2.78	0.26	62.31	193.77	10.65		
7AgL(G)	3	186.05*	234767.74***	148.02***	147.21***	1079.86***	1.71***	84.77	1728.13	38.54***		
R(Y)	4	74.57	40482.28	22.56	18.68	159.43**	0.77**	78.35	11376.96*	6.71		
Yx7AgL(G)	3	313.96**	140669.24***	26.40	12.08	69.54	0.97**	67.73	17225.15**	15.33		
Tx7AgL(G)	3	4.63	1424.65	5.12	3.52	10.21	0.17	101.85*	310.65	10.07		
TxYx7AgL(G)	3	132.47	86194.88*	72.47**	43.58*	7.32	0.30	30.01	2904.97	6.11		
Error	238	58.23	22279.13	12.62	13.18	31.91	0.18	36.79	3744.10	6.04		
R^2^		0.36	0.36	0.26	0.25	0.52	0.47	0.57	0.41	0.83		
		**(B**)										
		**B**	**HI**									
G	2	262.68	0.02***									
T	1	45.93	0.00									
R	2	297.15	0.02***									
TxG	2	440.10	0.01*									
7AgL(G)	3	1580.49**	0.00									
Tx7AgL(G)	3	865.71	0.00									
Error	129	325.00	0.00									
R^2^		0.16	0.33									
		**(C)**										
		**FLW**	**FLL**	**FLA**	**FL-1W**	**FL-1L**	**FL-1A**	**EL1**	**SNE1**	**SPNE1**	**SPS1**	**GYE1**
G	2	0.07	41.52*	114.32	0.01	164.19***	318.87***	46.68***	200.78	117.77***	9.23***	9.19***
Y	1	0.49***	19.20	452.92**	0.11*	35.27*	0.26	42.51***	8661.29***	0.00	48.91***	6.06**
T	1	0.72***	3.47	440.49**	1.23***	159.51***	1553.32***	6.00***	392.30	137.16***	1.49	0.90
YxG	2	0.39***	40.58*	155.84*	0.03	23.29*	74.39	2.15**	314.92	29.08***	3.37*	1.83
YxT	1	0.00	27.78	53.54	0.05	16.49	0.23	1.83*	10.77	24.47***	1.99	0.31
7AgL(G)	3	0.94***	125.29***	1248.63***	0.39***	91.05***	651.84***	3.34***	400.77	30.98***	3.15*	2.65**
R(Y)	4	0.04	2.16	39.23	0.07*	11.69	34.01	0.42	111.70	0.97	0.61	0.29
Yx7AgL(G)	3	0.06	2.17	23.59	0.07*	12.95	100.46*	0.55	846.02**	2.28	2.88*	2.06*
Tx7AgL(G)	3	0.02	1.06	18.05	0.04	7.18	53.41	0.12	61.18	1.17	0.24	0.52
TxYx7AgL(G)	3	0.01	7.37	14.92	0.01	3.26	3.83	0.11	156.28	0.44	0.75	1.11
Error	341	0.03	10.83	49.56	0.02	6.91	29.00	0.30	211.47	1.63	0.84	0.66
R^2^		0.36	0.16	0.25	0.33	0.31	0.35	0.67	0.17	0.57	0.28	0.19

Overall, the random year (Y) factor was significant for almost all traits, as expected from the rather different climatic conditions characterizing the two experimental years (see Supplementary Fig. S1 at *JXB* online). All genotypes underwent a longer growth cycle in 2010 compared to 2009 (see Supplementary Fig. S1 and Supplementary Table S1 at *JXB* online). For several traits (e.g. GY, TKW, TH, DTH, EL1, and SPNE1) the effect of the background genotype (G) was highly significant (Table 1), probably due to a low to intermediate heritability of yield per se and of its components, but also to residual heterogeneity in the NIRLs, particularly evident for R23-1. Line R23-1 not only underwent fewer BCs with the recurrent variety compared with the two other recombinants (see Materials and methods), but also harbours in the most proximal portion of its 7AgL segment an *Sd* gene(s) affecting its overall phenotypic stability (see Discussion). The third independent factor, ABA treatment (T), had no effect on any of the traits of the whole plant (Table 1A, 1B), and hardly any T×7AgL(G) or Y×T interaction was significant, indicating the absence of any causal relationship between 7AgL loci and response to ABA. The specific effects exerted by T on the treated main shoot (Table 1C) are described in a separate section (see ahead).

### Main genetic effects of the 7AgL segments on the whole plant yield traits

**Fig. 1.**
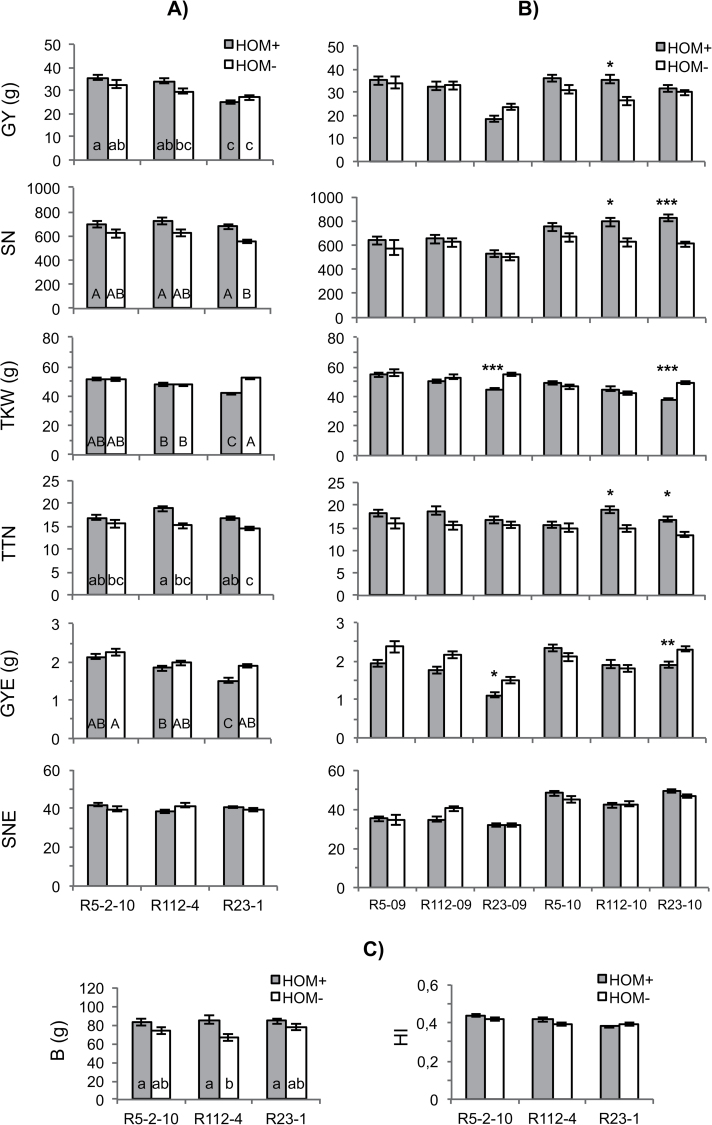
Yield-contributing traits recorded on the whole plant of homozygous durum wheat NIRLs (HOM+) and of their 7AgL non-carrier controls (HOM–) in 2009 and/or 2010: (A) 2009 + 2010; (B) 2009 versus 2010; (C) 2010 only. GY, grain yield per plant; SN, seed number per plant; TKW, thousand-kernel weight; GYE, grain yield per ear; SNE, seed number per ear; TTN, total productive tiller number; B, biomass per plant; HI, harvest index; letters correspond to ranking of groups after Tukey HSD test, capitals *P* <0.01, lower case *P* <0.05; *, ** and *** indicate significant differences after Tukey HSD test at *P* <0.05, *P* <0.01. and *P* <0.001, respectively; the absence of any mark indicates non-significance. R5, R5-2–10; R112, R112-4; R23, R23-1; 09, 2009; 10, 2010.

Interaction of G with 7AgL segment presence/absence [7AgL(G)] was in most cases highly significant, indicating a relevant contribution of loci present in the 7AgL segments to the expression of several traits (Table 1A, 1B). The 7AgL segments had a prevailingly positive effect across the two years (see Supplementary Table S1 at *JXB* online; Fig. 1), increasing most whole plant traits in the three durum recombinants (Fig. 1; see Supplementary Table S2 at *JXB* online), although significantly so only for SN of R23-1 (22%), for DTH of R112-4 (2.5%), and TTN and TTH of R112-4 and R23-1. For the latter two traits, increases were more prominent in R112-4 (average 25%) than in R23-1 (average 15%). In 2010, when differences for some whole plant traits between HOM+ and HOM– variants of each recombinant became more evident than in 2009 (see Supplementary Table S1 at *JXB* online; Fig. 1B), R112-4 HOM+ plants showed a highly significant increase in GY and SN (36.0% and 27.0%, respectively), paralleled by a considerably increased (28.3%) biomass (B), though not harvest index (HI) (Fig. 1C). For both these traits no difference was observed when HOM+ and HOM– plants of the other recombinants were compared.

On the other hand, the 7AgL segment of line R23-1 significantly decreased traits such as TKW (–20.1%) and the associated GYE (–20.5%) in both years (Fig. 1A, B). These trait reductions probably accounted for the lack of any significant difference in GY between R23-1 HOM+ and HOM– plants, despite the significantly higher SN associated with the presence of the 7AgL segment (Fig. 1).

### Main genetic effects of the 7AgL segments on the main shoot yield traits

**Fig. 2.**
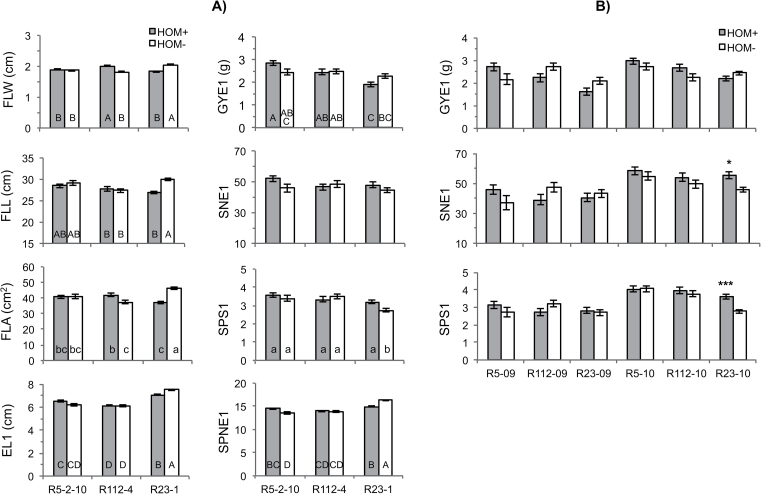
Yield-contributing traits recorded on the main shoot in 2009 and 2010 of homozygous durum wheat NIRLs (HOM+) and of their 7AgL non-carrier controls (HOM*-*): (A) 2009 + 2010; (B) 2009 versus 2010. FLW, flag leaf width; FLL, flag leaf length; FLA, flag leaf area; EL1, ear length; GYE1, grain yield per ear; SNE1, seed number per main shoot ear; SPS1, seeds per spikelet; SPNE1, spikelet number per ear; letters correspond to ranking of groups after Tukey HSD test: capitals, *P* <0.01, lower case, *P* <0.05; * and *** indicate significant differences at *P* <0.05 and *P* <0.001, respectively; the absence of any mark indicates non-significance. R5, R5-2–10; R112, R112-4; R23, R23-1; 09, 2009; 10, 2010.

The presence of 7AgL segments was associated with increases of almost all main shoot traits in R5-2–10 and R112-4 recombinants (Fig. 2), while the 7AgL segment present in the R23-1 recombinant negatively affected most traits. Compared with HOM– controls, FLW was significantly higher in R112-4 recombinant plants (+11%), and so was the derived FLA (+12.5%), while no change was observed for FLL, indicating the involvement of flag leaf width rather than length in the increase of leaf area. Dimensions of flag leaf and of flag leaf-1 were instead significantly decreased in R23-1 (Fig. 2A; see Supplementary Table S2 at *JXB* online).

On the other hand, SPNE1 was significantly increased (+7.7%) by the 7AgL segment of the R5-2–10 recombinant. Concomitantly, variation for EL1 was essentially significant (*P*=0.053), and in favour of HOM+ versus HOM– plants (Fig. 2A), while SNE1 and GYE1 showed no differences (Fig. 2A; see Supplementary Table S2 at *JXB* online). By contrast, SPNE1 and EL1 were significantly decreased (–8.4% and –6.0%, respectively) by the 7AgL segment in R23-1, while SPS1 was significantly increased in this recombinant across the two years (+17.0%), particularly in 2010 (+29.8%) together with SNE1 (+20.7%), confirming the higher potential for seed production of the R23-1 genotype (Fig. 2).

### Multivariate analysis for durum wheat NIRLs

To identify which traits contributed most to differences between the durum wheat genotypes, discriminant analyses (DA) were performed using the dataset of traits measured in both years, as well as data for 2010 alone, which included biomass. Using the presence/absence of the 7AgL segment of each recombinant as the grouping variable, the two analyses revealed that all groups had rather high percentages of correct classification (see Supplementary Table S3 at *JXB* online).

**Fig. 3.**
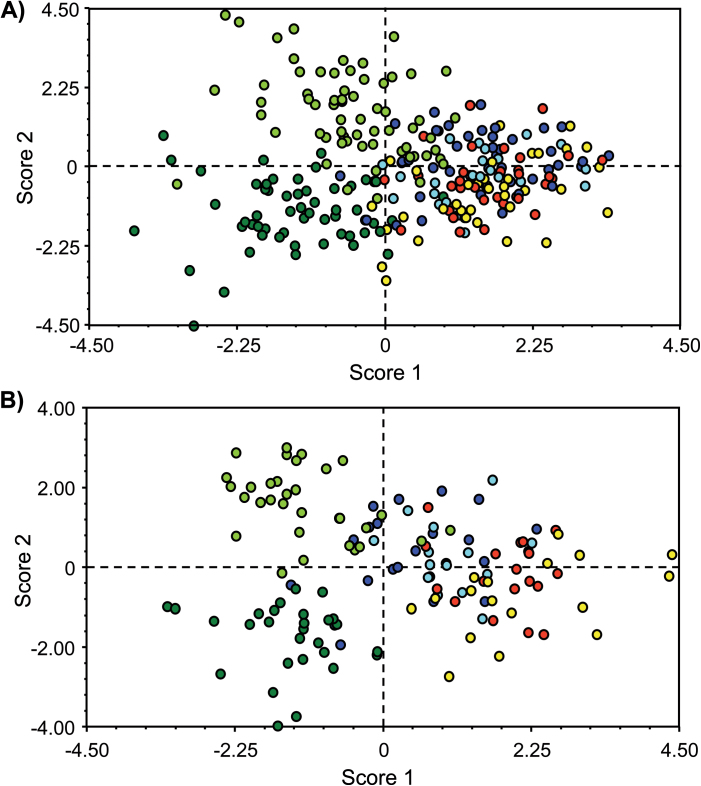
Discriminant analysis (DA) plot for homozygous durum wheat NIRLs (HOM+) and of their 7AgL non-carrier controls (HOM–) for traits recorded in (A) 2009 and 2010, and (B) 2010 only: dark blue, R5-2–10 HOM+; light blue, R5-2–10 HOM–; red, R112-4 HOM+; yellow, R112-4 HOM–; dark green, R23-1 HOM+; light green, R23-1 HOM–.

The first three discriminant functions (DFs) explained over 94% of the total variation between the groups for the 13 (2009–2010) and 14 (2010) variables (=traits) included in the model (see Supplementary Table S4 at *JXB* online). Grain yield (GY and GYE1), seed number (SN and SNE1), flag leaf width (FLW), and biomass (B) were the most important characters contributing to the first three DFs (see Supplementary Table S5 at *JXB* online), hence allowing discrimination between the observed groups. Scores of the first two DFs were then used for two-dimensional plotting of results (Fig. 3).

In both DAs the first DF divided the data into two main groups: one comprised the data points of line R23-1 (in green, Fig. 3), and the other the data points of lines R5-2–10 and R112-4. The traits that contributed the most to this discrimination were GY and GYE1 for the two-year dataset and B for the 2010 data analysis (see Supplementary Table S5 at *JXB* online). Groups could be further differentiated by the second DF, which led to separation of 7AgL segment carriers (HOM+) from non-carriers (HOM–), more evidently for R23-1 and less clearly in the other two lines (Fig. 3). The traits mostly responsible for the second DF separation were SN and SNE1 for the two-year data set, and GY and SN for 2010 (see Supplementary Table S5 at *JXB* online). These results are in line with the fact that the 7AgL segment of R23-1 recombinants significantly increases the number of seeds per plant and per spikelet, but concomitantly decreases seed weight (Fig. 1), and this significantly differentiates the R23-1 HOM+ group from the other two recombinants (Fig. 3). The discriminating power of B is a consequence of significant increases in tiller number shown by R112-4 and R23-1 recombinants, which led to increments in seed number and grain yield per plant, the main contributors to the observed classification.

### Yield and yield-related traits of bread wheat T4 line

The ANOVA–GLM applied to the two-year data set of the T4 NIL and of its Thatcher control (see Supplementary Table S6 at *JXB* online) explained 17–90% of variation for the whole plant traits and 38–85% for the traits measured on the treated main shoot (see Supplementary Table S1 at *JXB* online). ANOVA–GLM confirmed on these materials the highly significant effect of the random year (Y) factor on most traits (see Supplementary Table S6 at *JXB* online), indicating a different behaviour of both genotypes (with and without the 7AgL segment) under different climatic conditions. ABA treatment (T) alone exhibited a significant effect on some whole plant traits (GY, GYE, and TKW), unexpectedly increasing their values. On the other hand, only for these traits, the T x Y interaction also resulted statistically significant, but not T×7AgL, indicating that the observed significance can be largely attributed to the high variability of the mentioned traits across the two years (see Supplementary Table S1 at *JXB* online), rather than to a 7AgL effect.

For most of the whole plant traits, the effect of the 7AgL segment in line T4 [7AgL(G)] was not significant across years (see Supplementary Tables S2 and S6A and Supplementary Fig. S5A at *JXB* online). However, for TKW and eight traits of the main shoot the 7AgL(G) interaction was highly significant (see Supplementary Table S6A, C at *JXB* online), and a prevailingly negative effect of the 70%-long 7AgL segment on the majority of traits was observed (see Supplementary Table S2 at *JXB* online). In 2010, HI was also significantly lower (–5.5%) in T4 compared with Thatcher (see Supplementary Table S2 and, Supplementary Fig. S5B at *JXB* online). Overall, no advantages in productivity traits were observed to be associated with the T4 7AgL segment in the Thatcher background.

### Effect of ABA application and endogenous ABA content

**Fig. 4.**
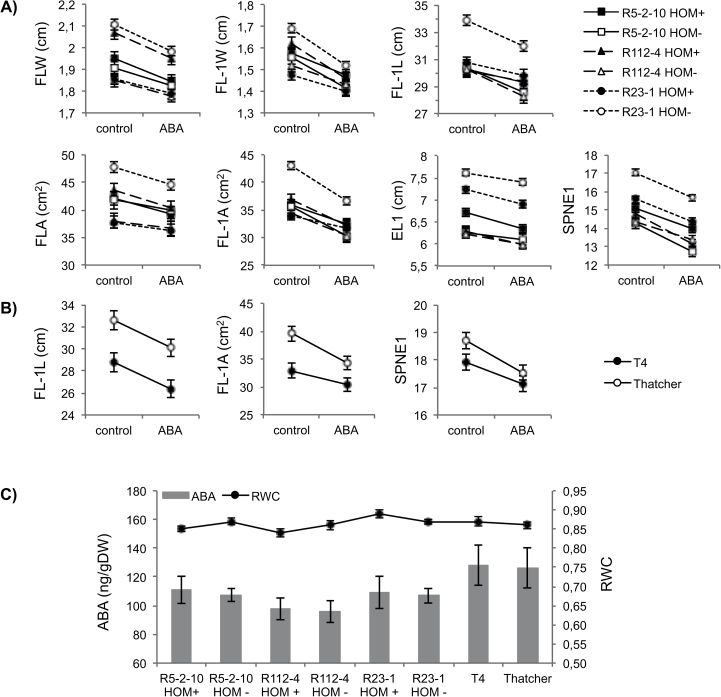
ABA and yield-contributing traits: effect of ABA treatment on yield-contributing traits of the main shoot of 7AgL-carrier (HOM+) and non-carrier (HOM–) genotypes of (A) durum wheat NIRLs and (B) bread wheat T4 line; (C) endogenous ABA content in spikes at the boot stage and relative water content (RWC) of the corresponding flag leaf. FLW, flag leaf width; FL-1W, flag leaf-1 width; FL-1L, flag leaf-1 length, FLA, flag leaf area; FL-1A, flag leaf-1 area; EL1, ear length; SPNE1, spikelet number per ear; DW, dry weight.

ABA treatment of the main shoot (T) of durum wheat genotypes significantly affected seven of the 11 traits (Table 1C) recorded on the treated shoot (i.e. FLW, FLA, FL-1W, FL-1L, FL-1A, EL1, and SPNE1). For all these traits ABA had a negative impact independently of 7AgL segment presence/absence (Fig. 4A), as confirmed by the absence of any significant T×7AgL(G) interaction (Table 1C). Although this second order interaction from ANOVA–GLM was significant for SNE, a trait referring to the whole plant (Table 1A), a subsequent Tukey HSD test revealed no significance for any of the pairwise comparisons between groups, which indicates that there is no real involvement of any of the alien segments in the response to ABA for SNE. The Tukey HSD test also showed the T×Y×7AgL(G) interaction to be non-significant for the treatment effect on SN, TTN, and TTH (Table 1A). On bread wheat line T4 and cv. Thatcher, the ABA treatment significantly affected only three traits (FL-1L, FL-1A, SPNE1) of the treated main shoot (see Supplementary Table S6C at *JXB* online; Fig. 4B), whose values were all decreased. For these traits, as observed for the durum wheat lines, ABA had no significant interaction with 7AgL segment presence/absence (see [T× 7AgL(G)] in Supplementary Table S6C at *JXB* online). Overall, at least in our experimental conditions, application of ABA had no differential effect on putative 7AgL-related yield loci.

Similarly, no significant difference associated with the presence of any 7AgL segment was observed for endogenous ABA content of juvenile spikes sampled from the main shoots of recombinant lines and corresponding controls in 2010, nor for RWC measured on the flag leaf of the same main shoot (Fig. 4C; see Supplementary Table S7 at *JXB* online).

## Discussion

### Comparing 7AgL effects in bread and durum wheat

To test the impact of loci located on the *Th. ponticum* 7AgL arm on wheat yield and yield-related traits, durum wheat recombinant lines, carrying differently sized 7AgL segments, were subjected to various experiments over two years. The same experiments included the bread wheat T4 translocation line, in which various yield-enhancing effects were originally associated with its 7AgL sizeable segment (Singh *et al.*, 1998; Reynolds *et al.*, 2001; Monneveux *et al.*, 2003; Tripathi *et al.*, 2005). Although a different homoeologue is involved in 7AgL transfers of durum and bread wheat recombinants (7AL versus 7DL, respectively), the shorter segments of durum recombinants represented a valid tool to ‘dissect’ the large 7AgL segment of line T4 into sub-regions and start delineating the relative contribution of loci located therein.

Under our experimental conditions, the T4 segment, incorporated into the old and unadapted bread wheat cv. Thatcher background, showed no positive effect on yield; instead, several yield and yield-related traits were depressed to a variable extent (see Supplementary Table S2 at *JXB* online). However, comparing the current evidence from the durum wheat recombinants with previous data on T4-derived bread wheat NILs, it clearly emerges that the magnitude of the effects shown by the durum wheat recombinants is much more conspicuous than that of the T4 genotypes. As an example, while the grain yield (GY) advantage of T4 derivatives over their controls ranged from 0.5% (Sibikeev *et al.*, 2000) to 16% (Singh *et al.*, 1998; Reynolds *et al.*, 2001; Monneveux *et al.*, 2003; Tripathi *et al.*, 2005), in the present study GY of R112-4 recombinant plants exceeded that of control plants by 36% (Fig. 1). Similarly, the reported 11–17.5% increase in grain number m^–2^ of T4 lines (Reynolds *et al.*, 2001; Monneveux *et al.*, 2003; Tripathi *et al.*, 2005) is considerably lower than that of the durum wheat recombinants analysed here (27% in R112-4 HOM+ and 36% in R23-1 HOM+ plants). Biomass also increased much less in T4 derivatives (8–10%, Reynolds *et al.*, 2001; Monneveux *et al.*, 2003; Tripathi *et al.*, 2005) than in the R112-4 durum wheat recombinant of this study (28%). Besides the quantitative differences, novel traits determined by 7AgL loci, notably tiller number per plant, have been highlighted for the first time using the durum recombinant NIRLs. Differences regarding the alien segment size, the recipient wheat chromosome, and the overall genome may all be contributing factors to such variation between bread and durum wheat 7AgL recombinants. In addition to this, durum wheat may benefit from an overall better responsiveness to variable growing conditions compared with bread wheat, at least in Mediterranean environments (Cossani *et al.*, 2011). In this type of environment, the background (species-specific) effect could, therefore, have reinforced the 7AgL-specific effects observed in our durum recombinant genotypes. In fact, the presence of 7AgL segments did not determine the marked negative effects on yield of these recombinants in the much drier and warmer 2009 season (see Supplementary Fig. S1 at *JXB* online), while T4 lines underwent significant yield reductions in water-stress conditions (16%, Singh *et al.*, 1998; 21.5%, Monneveux *et al.*, 2003).

### Structural/functional ‘dissection’ of 7AgL segments in durum wheat recombinant lines

**Fig. 5.**
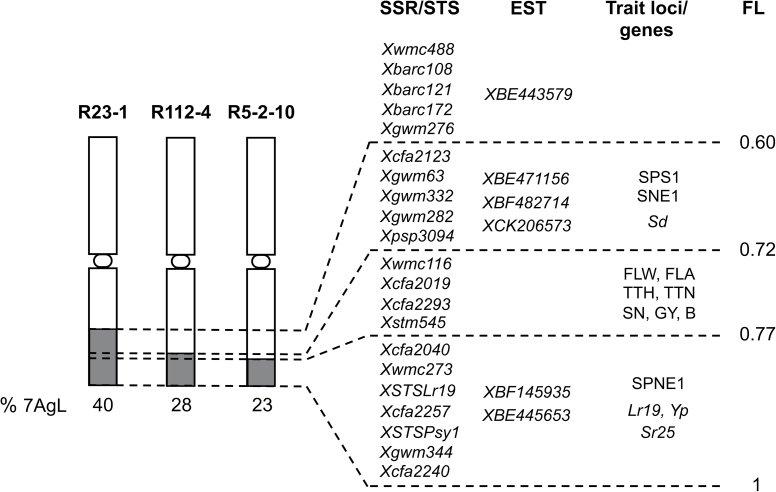
Cytogenetic maps of the 7A-7Ag recombinant chromosomes representing the durum wheat–*Th. ponticum* NIRLs used in the present study (adapted from Ceoloni *et al.,* 2005, 2014), with assignment to defined 7AgL regions of markers (SSR, simple sequence repeats or microsatellites; STS, sequence tagged sites; EST, expressed sequence tags), known genes (*Lr19*, *Sr25*, *Yp*, *Sd*, see text), and putative loci for yield-contributing traits (SPS1, seed number per spikelet; SNE1, seed number per ear; FLW, flag leaf width; FLA, flag leaf area; TTH, tiller number at heading; TTN, total productive tiller number; SN, seed number per plant; GY, grain yield per plant; B, biomass per plant; SPNE1, spikelet number per ear). White background, 7A, grey background, 7AgL. FL, fractional arm length of the distance from the centromere, from 0=centromere to 1=telomere.

On the basis of significant differences for the traits examined between HOM+ and HOM– plants of the three durum wheat NIRLs, sub-division of the 7AgL chromatin spanning 40% of the recombinant 7AL arm into functionally different regions was possible. As a result of this ‘introgression mapping’, the genetic control of 10 of the 20 traits analysed could be associated with defined 7AgL portions, hence allowing the drawing of a tentative structural-functional map of the 7AL-7AgL region in question (Fig. 5). From telomere to centromere, the control of spikelet number per ear was assigned to the most distal 23% of 7AgL, as the trait was more markedly expressed in HOM+ plants of the R5-2–10 line than in the other two recombinants (Fig. 2); genetic determinants of flag leaf width, tiller number, seed number, and grain yield per plant, as well as biomass, appeared to be concentrated in the proximally adjacent 5% alien chromatin, included in lines R112-4 and R23-1; finally, loci for seed number per ear and per spikelet appeared to reside in the 7AgL portion present in R23-1 only, i.e. in the 7AgL fraction occupying 12% of the 7AL recombinant arm and extending beyond the R112-4 breakpoint.

Clustering of loci involved in the control of multiple source and sink traits, as observed in this study, is not unusual in cereal genomics. In wheat, for example, a QTL for flag leaf width was co-located with those for grain number and grain yield (Quarrie *et al.*, 2006), and a QTL for spike number with QTLs for spike length and grain number per ear (Deng *et al.*, 2011). In rice, several studies reported the identification of QTL regions affecting concomitantly different panicle traits (Tian *et al.*, 2006), also in association with other yield-related traits, (Xie *et al.*, 2008, Wang *et al.*, 2012*b*, and references therein).

The existence of multiple loci affecting yield traits along the 7AgL regions introgressed into wheat was a likely hypothesis, considering the several loci for such traits reported on wheat homoeologous arms, notably 7AL (see Introduction), and the substantial colinearity emerged from the wheat 7AL versus *Th. ponticum* 7AgL genetic map comparison (Kuzmanović, 2011; Ceoloni *et al.*, 2014). The wheat 7AL region where Quarrie *et al.* (2005) placed a strong yield QTL, mainly affecting grain number per spike, is of particular interest for our 7AL versus 7AgL comparison. That region, between SSR marker loci *Xpsr3094* and *Xwmc273*, could be better resolved by use of the durum wheat–*Th. ponticum* recombinant lines. Figure 5 shows that *Xpsr3094* resides in the chromosomal region delimited by the 7AL-7AgL BPs of R112-4 and R23-1 recombinants, i.e. between FL (fractional arm length of the BP distance from the centromere, from 0=centromere to 1=telomere) 0.72 and 0.60 of the recombinant arm, while *Xwmc273* is situated in the last 23% of the arm, within the 7AgL segment present in line R5-2–10.

Subsequent work by Quarrie *et al.* (2006; unpublished data) showed the 7AL yield/grain number per ear QTL to be at or proximal to *Xpsr3094*, suggesting that it might correspond to a gene controlling flag leaf width and chlorophyll content, thereby indirectly affecting the amount of assimilates transferred into the spike. The significant increase of seed number per ear (SNE1) and per spikelet (SPS1) exclusively observed in the R23-1 recombinant in this study, leads to the assignment of a putative gene(s) controlling seed number per ear to the 12% 7AgL region differentiating R23-1 from R112-4 lines and including locus *Xpsr3094* (Fig. 5). This would confirm the hypothesis of the presence on that 7AgL portion of a locus for grain number homoeologous to that identified on 7AL by Quarrie *et al.* (2005). As often observed (Slafer *et al.*, 1996; Acreche and Slafer, 2006), the increased seed number was negatively correlated with seed weight in the R23-1 recombinant, which suffered from a 20% reduction in TKW (Fig. 1A). However, this was not the case for the R112-4 recombinant which, in 2010, also showed significantly increased seed number per plant (SN) without alteration in TKW. Since there is evidence in wheat of separate genetic control of seed number and seed weight (Ji *et al.*, 2010), one could hypothesize that the R23-1 recombinant contains two loci on its 7AgL segment between FL 0.72 and 0.60, one increasing seed number per spike, and the other decreasing seed weight. The 7AgL region in question corresponds to that of 7AL identified as containing QTL for TKW, as reported by Huang *et al.* (2004) (associated with locus *Xgwm282*; Fig. 5) and by Raman *et al.* (2009) (between loci *Xgwm332* and *Xstm545*; Fig. 5).

On the other hand, the R112-4 recombinant had a significant involvement in the control of FLW and, consequently of FLA (11% and 12.5% increase, respectively), which was not observed in the other two recombinants. A larger flag leaf is likely to increase whole plant photosynthetic rate and hence grain yield, a positive relationship often detected in wheat (Simpson, 1968; Monyo and Whittington, 1973; Quarrie *et al.*, 2006; Snape *et al.*, 2007; Bennett *et al.*, 2012), rice (Yue *et al.*, 2006; Wang *et al.*, 2012*b*), and barley (Yap *et al.*, 1972; Xue *et al.*, 2008). Flag leaf dimensions were also differentially expressed in R23-1 HOM+ versus HOM– plants, although the results were markedly decreased when the 7AgL segment was present (Fig. 2A). Based on this evidence, it can be hypothesized that a genetic determinant of flag leaf development might be located in the 7AgL portion present in R112-4 and shared with R23-1 but absent in R5-2–10 (Fig. 5). This interpretation is not consistent with the hypothesis of Quarrie *et al.* (2006) of a rather tight FLW-*Xpsr3094* association, although their genetic map had poor resolution in that region of 7AL. Therefore, either there is more than one locus controlling the observed variation in source (flag leaf) and sink (grain number) traits in that 7AgL region, and the low resolution of the corresponding 7AL region in bread wheat (Quarrie *et al.*, 2005) was unable to show it, or there are inherent differences between the genetic/physical make-up of the 7AL and 7AgL arms. Nevertheless, the two hypotheses need not necessarily be mutually exclusive.

Tiller number, a trait of particular importance in maximizing yield (Elhani *et al.*, 2007), also seems to have a locus along 7AgL located between FL 0.77 and 0.72 (Fig. 5) of the 7AL-7AgL recombinant arm(s). Across the two seasons, tiller number, both at heading (TTH) and at maturity (TTN) (Fig. 5), was significantly affected by the presence of 7AgL chromatin shared by R112-4 and R23-1 durum wheat recombinants, the former showing a more marked increase compared with the latter. No effect on tiller number was observed in R5-2–10, which indicated that the underlying genetic factor(s) is located in the 7AgL region common to R112-4 and R23-1 but absent in R5-2–10. This chromosomal region seems to correspond to that identified in bread wheat by Heidari *et al.* (2011) as harbouring a QTL for spikes m^–2^, and flanked by *Xgwm282* and *Xwmc116* marker loci on 7AL (Fig. 5), which could thus include homoeoloci in both 7AL and 7AgL. Although not all tillers produce spikes at maturity, in general, tiller productivity is proportional to the plant capacity to capture resources and hence yield more (Sadras and Slafer, 2012). In fact, the trait was found to be highly correlated with yield across environments in a number of studies (Huang *et al.*, 2003; Kumar *et al.*, 2007; Narouka *et al.*, 2011). In addition, the increase in tiller number was reported not to imply a yield penalty for durum wheat grown in Mediterranean environments but, instead, to ensure a minimum yield even under water-stressed conditions (Elhani *et al.*, 2007). The 0.77–0.72 7AgL region identified here appears to be involved in promoting early vegetative growth (TTH) and in maintaining a high number of productive tillers at maturity (TTN), which would have contributed to the increase of final grain yield (Figs. 1, 5). Data from 2010 showed that the increase of tiller number in the 7AgL-carrier lines when compared to their controls was also responsible for increased biomass, without altering the plants’ harvest index (Fig. 1C). This was particularly evident for the R112-4 recombinant which, in 2010, concomitantly showed a significantly higher B, GY, and SN.

As to the 7AgL segment shared by the three durum recombinant lines, spanning the most distal 23% of the 7AL-7AgL arm, the positive effect on SPNE1 associated with this segment was much better expressed in the absence of additional 7AgL chromatin (Fig. 2). This suggests the possible existence in the more proximal 7AgL portion of other alien genes that mask its expression. The locus controlling SPNE1 falls in the chromosomal interval on 7AL flanked by markers *Xcfa2019* and *Xcfa2257* (Fig. 5), where a minor QTL for fertile spikelet number was previously located in bread wheat (Ma *et al.*, 2007). Quarrie (unpublished data) also found a frequent effect on spikelet number per ear in a bread wheat mapping population in the distal portion of 7AL containing the locus *Xcfa2019*. Thus, 7AgL could well carry a possible orthologue at a roughly colinear position.

### Negative impacts of certain 7AgL portions

Concomitantly with the highly positive traits of seed number per ear and per spikelet associated with the most proximal portion of 7AgL introduced in the durum wheat–*Th. ponticum* recombinant R23-1 (Fig. 5), many other traits related to the uppermost leaves and spike/seed characteristics (see Supplementary Table S2 at *JXB* online; Fig. 2A), were markedly depressed in the R23-1 recombinant. Such effects may be a general consequence of linkage drag, frequently associated with sizeable alien introgressions, particularly at the tetraploid level (Ceoloni and Jauhar, 2006). However, a number of abnormalities expressed by R23-1 plants seem to have a more specific driving factor (Grossi *et al.*, 2009; Ceoloni *et al.*, 2014). In fact, not only the R23-1 recombinant chromosome, and so-derived chromosome types retaining the most proximal 7AgL portion of the its 7AgL segment, showed reduced male transmission compared with that of the R112-4 and R5-2–10 types (Ceoloni *et al.*, 2005, 2014), but was also found to determine a number of frequently associated abnormalities, including aberrant mitotic pollen divisions, seed set reduction, and/or seed shrivelling, and, sometimes, abnormal plant phenotypes (Grossi *et al.*, 2009; Ceoloni *et al*., unpublished data). Thus, the 0.72-0–60 7AgL portion of the R23-1 segment could correspond to the physical location of one of possibly multiple segregation distortion (*Sd*) factors (Fig. 5) suggested to be present along the *Th. ponticum* 7Ag chromosome (Prins and Marais, 1999; Cai *et al.*, 2011; Ceoloni *et al.*, 2014). Segregation distortion has also been observed in bread wheat backgrounds containing the 7D chromosome with the T4 translocation, or modified forms of it (Prins and Marais, 1999; Marais *et al.*, 2001). However, phenotypic drawbacks in T4 genotypes, where reported, were generally associated with unfavourable environmental conditions, notably drought (Singh *et al.*, 1998; Monneveux *et al.*, 2003). While it is plausible that the durum wheat background shows an overall higher sensitivity to *Sd*-linked effects, in the present study a nearly 20% reduction in the size of the two uppermost leaves of the main shoot was observed in both R23-1 HOM+ and T4 plants (see Supplementary Table S2 at *JXB* online). The decreased surface of such leaves, which represent the primary source of carbohydrates for the spike during grain filling, might have had a negative effect on grain weight, leading to the 20% and 7.5% lower TKW in R23-1 and T4 plants than in their controls. A 16.2% decrease in grain yield of the main shoot (GYE1) was also detected in T4 plants (see Supplementary Table S2 at *JXB* online). These results suggest that genetic determinant(s) negatively affecting, in a direct or indirect manner, the grain-filling process may be located in the 7AgL chromatin present in both T4 and R23-1 lines and absent from the two other durum recombinants. Whether such gene(s) correspond(s) to an *Sd* factor or it is an *Sd* pleiotropic effect or, alternatively, it is simply associated with it in the 7AgL portion proximal to the R112-4 breakpoint, cannot be established with the currently available information and genetic materials.

### Relationship between yield traits of the main shoot and ABA

Since ABA concentration increases in most organs of water-stressed plants (Ahmadi and Baker, 1999; Wittenmayer and Merbach, 2005), it is often regarded as a growth inhibitor. In wheat, in particular, fertility and grain-filling rate are significantly and inversely dependent on the level of endogenous ABA during ear development (Cao *et al.*, 2000; Lin *et al.*, 2007; Liu *et al.*, 2013). The wheat ear tends to act as a sink for ABA translocated from leaves, a process largely conditioned by the water status of the flag leaf and floral organs (Morgan and King, 1984; Westgate *et al.*, 1996). Comparable RWC values of the flag leaf of HOM+ versus HOM– plants of each recombinant observed in the present study (Fig. 4C) indicate that plants did not suffer from water stress at the booting stage of development and that the endogenous ABA measured represents the intrinsic amount of the hormone produced by a given genotype. The results obtained did not confirm the only previous observation of this kind on T4 (Reynolds *et al.*, 2008), which reported lower levels of ABA in T4 NILs, displaying a higher number of grains per spike compared with their controls, nor did they reveal any significant difference in ABA content associated with the presence of 7AgL segments in the durum wheat genotypes (Fig. 4C).

An absence of 7AgL segment-dependent differences was also revealed by an analysis of fertility parameters of the main shoot treated with exogenous ABA. The treatment was performed at the ‘double ridge’ stage of ear development, when the leaf primordia finish and the reproductive organ primordia (spikelets, florets) begin to differentiate; so the effect of ABA was expected to be significant on these plant organs and yield traits determined by them, including flag leaf width and spikelet and seed number (Wang *et al.* 2001). In fact, the ABA treatment generally had a depressing effect on the dimensions of the two uppermost leaves, ear length, and spikelet number (Fig. 4A, B), but this was apparently non-7AgL-dependent and had no impact on the final grain yield. In fact, although ABA action is systemic in plants, no significant variation for whole plant yield-contributing traits was found to be due to ABA treatment (T). Thus, so far it cannot be hypothesized that an ABA-responsive mechanism is involved in the expression of 7AgL yield-contributing QTL as found for the yield QTL linked to the *Xpsp3094* locus on bread wheat 7AL (Quarrie *et al.*, 2007).

## Conclusions and future work

Altogether, the 28%-long 7AgL segment introgressed into 7AL of the R112-4 recombinant carried multiple beneficial effects on yield traits with no apparent detrimental side-effects on the tetraploid recipient genome. Moreover, the simultaneous presence of other genes of agronomic relevance, such as *Lr19*, *Sr25*, and *Yp* (Fig. 5), makes the R112-4 recombinant a highly valuable source that can be readily deployed in durum wheat breeding programmes, at least in Mediterranean environments. On the other hand, the extra 7AgL portion beyond the R112-4 breakpoint present in the R23-1 recombinant was associated with various negative aspects, possibly due to the presence of an *Sd* gene. This and/or other unfavourable genetic factors hamper the exploitation in durum wheat of the 7AgL segment specific to line R23-1. As hexaploid bread wheat is expected not to suffer to the same extent from similar constraints, the engineering of bread wheat 7AL with the 40%-long 7AgL *Th. ponticum* segment, and also with the two shorter segments characterized in the course of the present study, has recently been undertaken. This will allow their separate effects in a bread wheat background to be assessed better when compared with those of the entire T4 translocation, of which they represent fractions, and with those observed in durum wheat. Future work will focus on bigger scale and multi-location field trials with the same durum wheat NIRLs as well as on development and use of further recombinant lines, in order to narrow down the critical regions for yield-related traits. A better marker coverage of these regions is a further objective, particularly by exploiting the high-throughput technology, also recently developed in durum wheat (van Poecke *et al*., 2013), for finer mapping and characterization as well as for MAS breeding and eventual cloning of target loci.

## Supplementary data

Supplementary data can be found at *JXB* online.

Supplementary Fig. S1. Daily precipitations, maximum (Tmax) and minimum (Tmin) temperatures, and radiation during 2009 and 2010 as retrieved from the meteorological station of the experimental farm of the University of Tuscia, Viterbo, Italy. S, sowing date; GH, transfer of plants to glasshouse; ABA, the week of ABA treatment; F, transfer of plants to field; Hd, heading; H, harvest.

Supplementary Fig. S2. Dissection of a plant prior to ABA treatment. (A) Entire plant look; (B) dissected leaves 1–7; (C) dissected leaves 8–10 at stereoscope; (D) early double ridge stage (Zadoks 20, Zadoks *et al.*, 1974).

Supplementary Fig. S3. Application of ABA onto leaves. (A) Laying down of a solution drop; (B) leaf perforation by fine needle.

Supplementary Fig. S4. Wheat ear at the boot stage of development (Zadoks 45, Zadoks *et al.*, 1974).

Supplementary Fig. S5. Yield-contributing whole plant traits recorded for T4 bread wheat NIL and its control cv. Thatcher. (A) 2009 + 2010; (B) 2010 only. GY, grain yield per plant (g); SN, seed number per plant; TKW, thousand-kernel weight (g); GYE, grain yield per ear (g); SNE, seed number per ear; TTN, total productive tiller number; B, biomass per plant (g); HI, harvest index; letters correspond to ranking of groups after Tukey HSD test at *P* <0.05; the absence of any letter indicates non-significance.

Supplementary Table S1. Phenotypic values of yield-related traits in durum and bread wheat recombinant lines and their controls recorded in 2009 and 2010.

Supplementary Table S2. Two-year means and standard errors (SE) as from ANOVA-GLM analyses for yield-contributing traits of the durum and bread wheat 7AgL recombinant lines and their respective controls.

Supplementary Table S3. Classification matrix (cases in row categories classified into columns) of discriminant analysis (DA) applied to HOM+ durum wheat NIRLs and to their HOM– controls for traits recorded in (A) 2009 and 2010, (B) 2010 only.

Supplementary Table S4. Eigen values, total variance, and cumulative variance of discriminant analysis (DA) for traits of HOM+ and HOM– plants of durum wheat NIRLs for (A) 2009 and 2010, (B) 2010 only; DF, discriminant function.

Supplementary Table S5. Canonical discriminant functions (DFs) standardized by within variances of discriminant analysis (DA) for traits of HOM+ and HOM– plants of durum wheat NIRLs for (A) 2009 and 2010, (B) 2010 only. Values in bold indicate traits mainly contributing to the classification at a specific DF.

Supplementary Table S6. Mean squares from ANOVA–GLM for yield and yield-related traits of the bread wheat T4 NIL and its control cv. Thatcher determined in 2009 and/or 2010. (A) Whole plant traits in 2009 + 2010; (B) whole plant traits in 2010; (C) main shoot traits in 2009 + 2010. GY, grain yield per plant (g); SN, seed number per plant; TTN, total productive tiller number; TTH, tiller number per plant at heading; TKW, thousand-kernel weight (g); GYE, grain yield per ear (g); SNE, seed number per ear; TH, plant height (mm); DTH, days to heading; FLW, flag leaf width (cm); FLL, flag leaf length (cm); FLA, flag leaf area (cm^2^); FL-1W, flag leaf-1 width (cm); FL-1L, flag leaf-1 length (cm); FL-1A, flag leaf-1 area (cm^2^); EL1, ear length (cm); SNE1, seed number per main shoot ear; SPNE1, spikelet number per main shoot ear; SPS1, seeds per spikelet; GYE1, grain yield per main shoot ear (g); B, biomass (g); HI, harvest index; *, **, *** indicate significant *F* values at *P* <0.05, *P* <0.01, and *P* <0.001, respectively.

Supplementary Table S7. ANOVA–GLM summary table for endogenous ABA content in juvenile spikes of (A) durum wheat NIRLs and (B) bread wheat T4 NIL, and for corresponding relative water content (RWC) of (C) durum wheat NIRLs and (D) bread wheat T4 NIL; SS, sum of squares; MS, mean squares.

Supplementary Data

## Abbreviations:

ABA: abscisic acid
ANOVA: analysis of variance
B: dry biomass of the whole above-ground part of the plant
BC: backcross
CV: coefficient of variation
DA: discriminant analysis
DTH: number of days to heading
EL1: ear length of the main shoot
FL-1A: flag leaf-1 area
FL-1L: flag leaf-1 length
FL-1W: flag leaf-1 width
FLA: flag leaf area
FLL: flag leaf length
FLW: flag leaf width
GLM: general linear model
GY: grain yield per plant
GYE: grain yield per ear
GYE1: grain yield per ear of the main shoot
HI: harvest index
HOM–: homozygous non-carrier of given alien segment
HOM+: homozygous carrier of given alien segment
MAS: marker assisted selection
NIL: near-isogenic line
NIRL: near-isogenic recombinant line
QTL: quantitative trait locus/loci
RIA: radioimmunoassay
RWC: relative water content
SN: seed number per plant
SNE: seed number per ear
SNE1: seed number per ear of the main shoot
SPNE1: spikelet number per ear of the main shoot
SPS1: seeds per spikelet of the main shoot
TH: total plant height
TKW: thousand-kernel weight
TTH: tiller number per plant at heading
TTN: total productive tiller number.
