# Supplementary material for: Structural–functional dissection and characterization of yield-contributing traits originating from a group 7 chromosome of the wheatgrass species Thinopyrum ponticum after transfer into durum wheat
Source: J Exp Bot. 2013 Dec 6;65(2):509–25. doi: 10.1093/jxb/ert393 (PMC3904708; doi:10.1093/jxb/ert393)
Supplement: Supplementary Data [file supp_65_2_509__index.html]

Structural–functional dissection and characterization of yield-contributing traits originating from a group 7 chromosome of the wheatgrass species Thinopyrum ponticum after transfer into durum wheat — Supplementary Data 

# Structural–functional dissection and characterization of yield-contributing traits originating from a group 7 chromosome of the wheatgrass species *Thinopyrum ponticum* after transfer into durum wheat

## Supplementary Data

Data files

**Files in this Data Supplement:**

- Supplementary Data - Supplementary Data
