# Supplementary material for: Structural–functional dissection and characterization of yield-contributing traits originating from a group 7 chromosome of the wheatgrass species Thinopyrum ponticum after transfer into durum wheat
Source: J Exp Bot. 2013 Dec 6;65(2):509–25. doi: 10.1093/jxb/ert393 (PMC3904708; doi:10.1093/jxb/ert393)
Supplement: Supplementary Data [file supp_ert393_Supplementary_Data_corrected_ert393.pdf]

Journal of Experimental Botany

Structural-functional dissection and characterization of yield-contributing traits originating from a group 7 chromosome of the wheatgrass species *Thinopyrum ponticum* after transfer into durum wheat

Ljiljana Kuzmanović, Andrea Gennaro, Stefano Benedettelli, Ian C. Dodd, Stephen A. Quarrie, Carla Ceoloni

Supplementary Figures

Supplementary Fig. S1. Daily precipitations, maximum (Tmax) and minimum (Tmin) temperatures and radiation during 2009 and 2010 as retrieved from the meteorological station of the experimental farm of the University of Tuscia, Viterbo, Italy. S = sowing date; GH = transfer of plants to glasshouse; ABA = the week of ABA treatment; F = transfer of plants to field; Hd = heading; H = harvest.

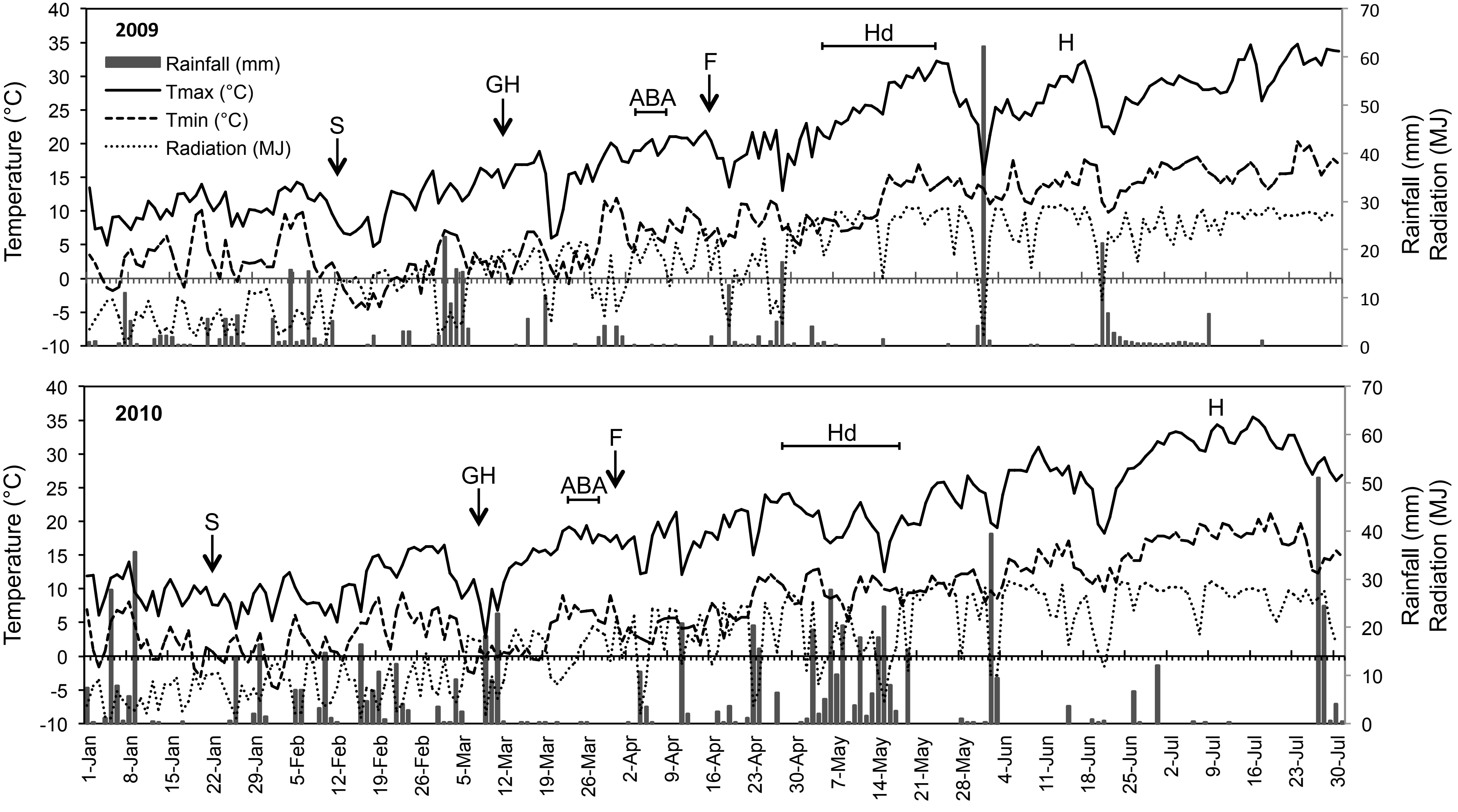

Supplementary Fig. S2. Dissection of plant prior to ABA treatment: A) entire plant look, B) dissected leaves 1-7, C) dissected leaves 8-10 at stereoscope, D) early double ridge stage (Zadoks 20, Zadoks *et al*., 1974).

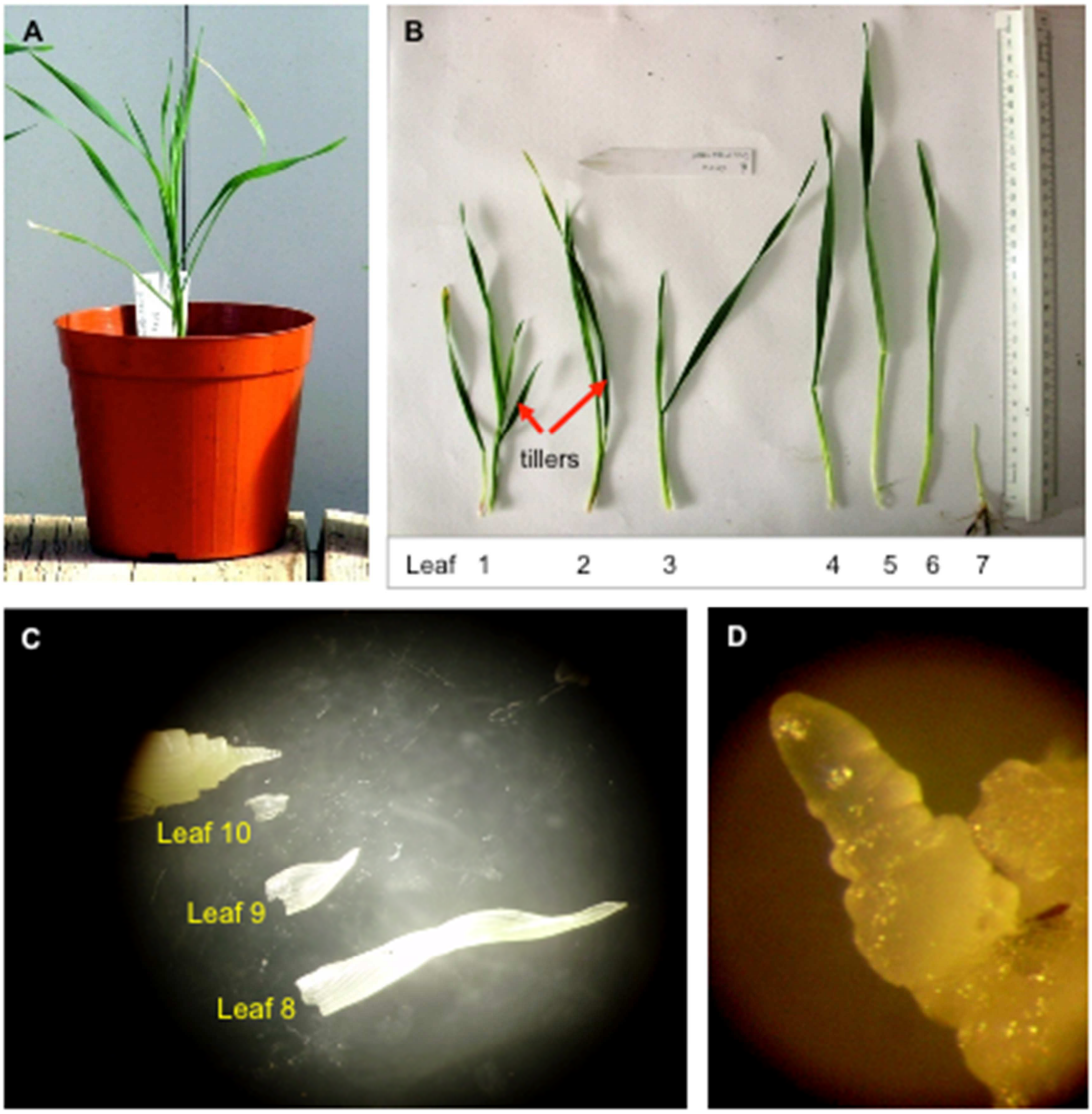

Supplementary Fig. S3. Application of ABA onto leaves: A - laying down of a solution drop, B – leaf perforation by fine needle.

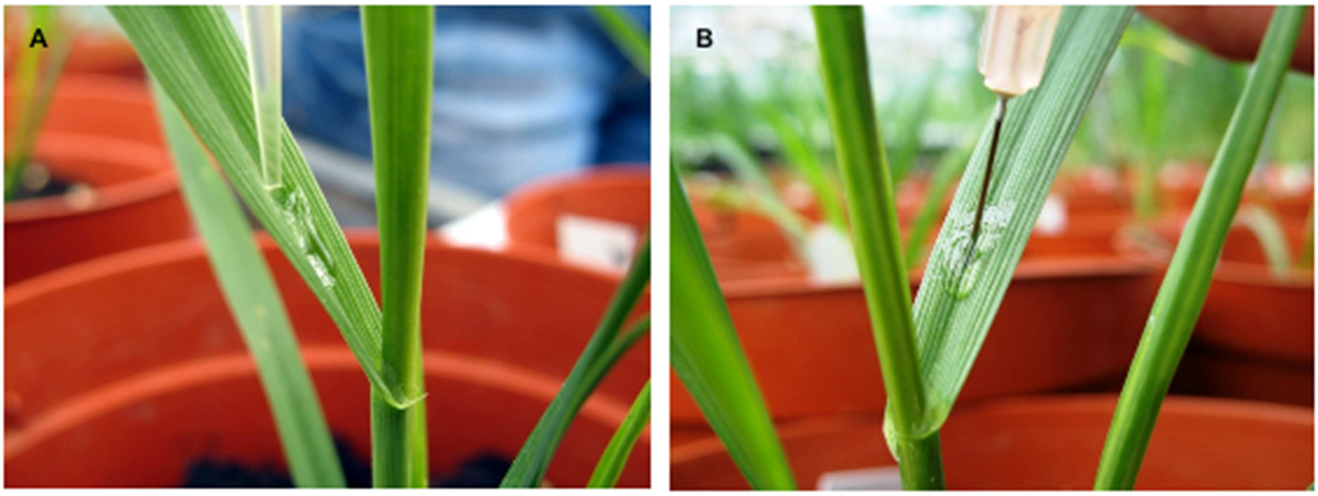

Supplementary Fig. S4. Wheat ear at the boot stage of development (Zadoks 45, Zadoks *et al.*, 1974).

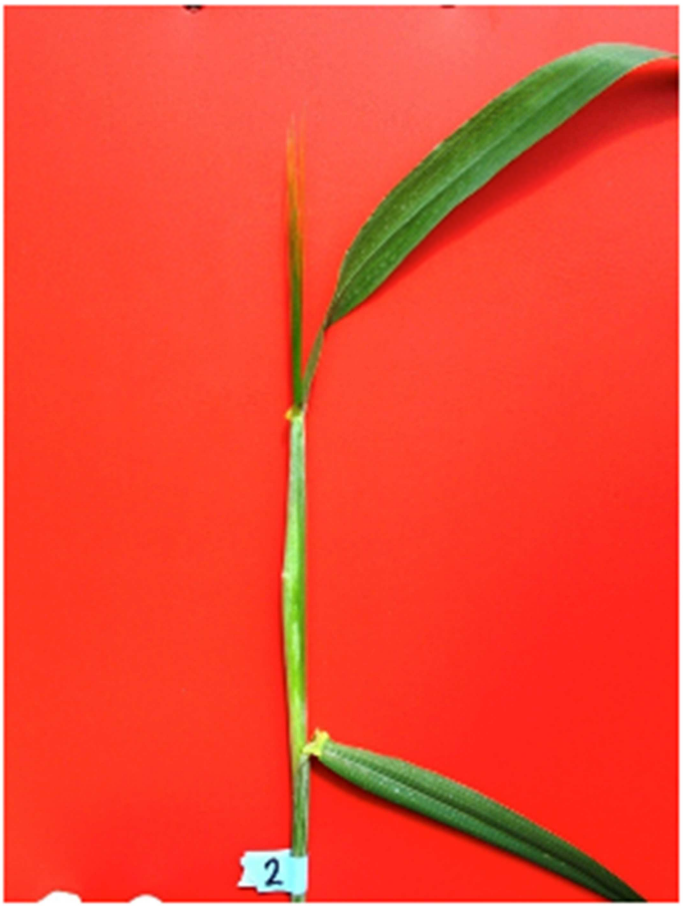

Supplementary Fig. S5. Yield-contributing whole plant traits recorded for T4 bread wheat NIL and its control cv. Thatcher (A = 2009+2010; B = 2010 only); GY: grain yield per plant (g), SN: seed number per plant, TKW: thousand-kernel weight (g), GYE: grain yield per ear (g), SNE: seed number per ear; TTN: total productive tiller number, B: biomass per plant (g), HI: harvest index; letters correspond to ranking of groups after Tukey HSD test at $P<0.05$; absence of any letter indicates non significance.

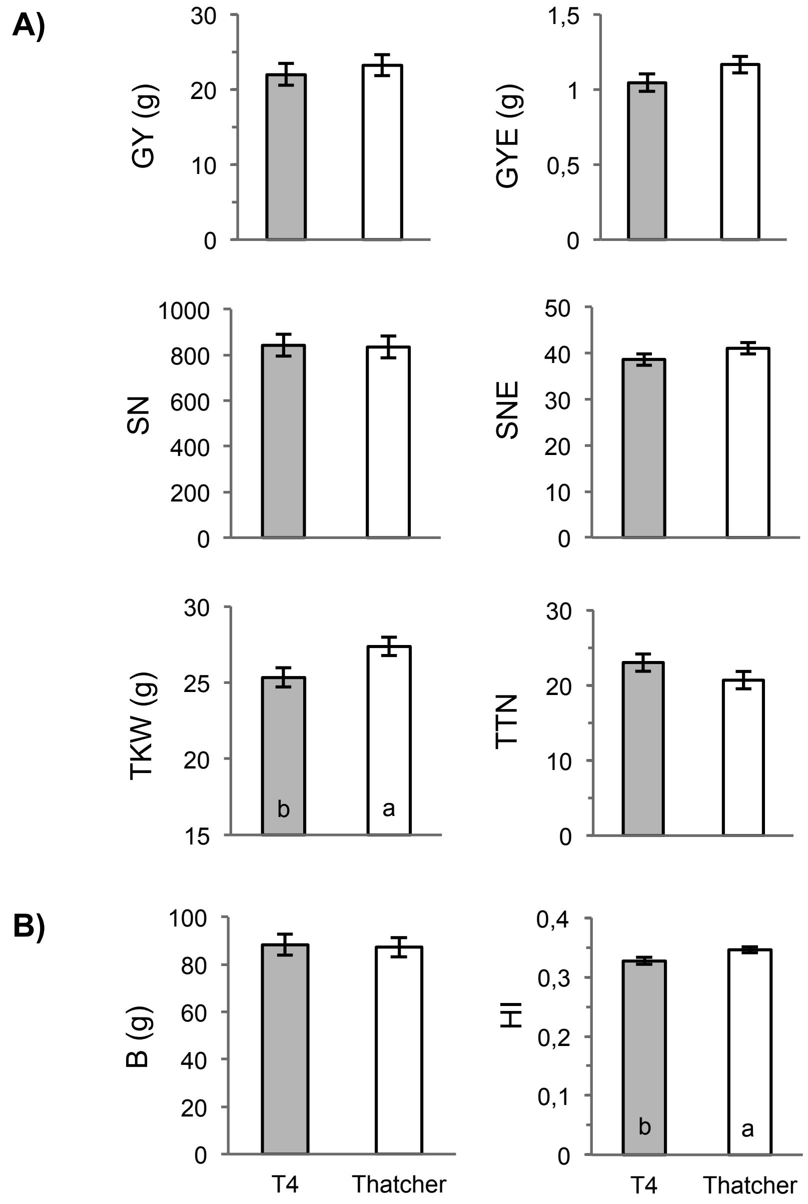

Supplementary Tables

Supplementary Table S1. Phenotypic values of yield-related traits in durum and bread wheat recombinant lines and their controls recorded in 2009 and 2010.

Trait[1]	Item	2009								2010							
		R5-2-10 HOM+	R5-2-10 HOM-	R112-4 HOM+	R112-4 HOM-	R23-1 HOM+	R23-1 HOM-	T4	Thatcher	R5-2-10 HOM+	R5-2-10 HOM-	R112-4 HOM+	R112-4 HOM-	R23-1 HOM+	R23-1 HOM-	T4	Thatcher
GY	Mean	35.17	34.69	32.65	32.92	19.01	24.66	16.34	17.28	36.37	31.03	35.77	26.15	31.40	29.94	28.83	30.11
	Minimum	18.80	22.31	26.15	20.29	9.94	10.09	7.78	8.76	25.02	18.91	24.10	12.45	21.51	16.87	17.55	22.28
	Maximum	49.92	44.51	43.24	51.37	32.59	42.88	27.56	31.30	47.09	42.18	49.18	39.67	46.85	46.26	42.60	46.80
	CV[2]	0.22	0.20	0.16	0.25	0.35	0.38	0.47	0.52	0.18	0.28	0.22	0.29	0.24	0.26	0.23	0.24
	SE[3]	1.77	2.25	1.26	1.93	1.29	1.71	2.90	3.43	1.34	2.19	1.83	1.76	1.39	1.28	1.50	1.51
SN	Mean	641.70	589.50	652.00	619.57	535.79	517.26	753.46	747.68	753.21	663.49	798.49	623.77	821.15	604.44	949.79	930.17
	Minimum	475.60	451.13	473.96	397.73	307.54	268.88	577.20	466.98	536.52	410.43	596.86	378.12	542.75	337.07	580.72	585.98
	Maximum	929.50	692.04	896.44	847.17	810.36	874.32	937.76	1049.44	977.34	878.16	1180.65	897.81	1230.20	930.92	1499.15	1420.98
	CV	0.18	0.12	0.20	0.20	0.29	0.31	0.20	0.28	0.17	0.25	0.20	0.27	0.23	0.25	0.24	0.24
	SE	26.30	27.45	30.95	29.77	30.23	29.58	56.92	78.03	27.61	40.61	36.67	38.20	35.01	24.70	52.66	47.69
TKW	Mean	54.55	57.32	50.64	53.00	45.37	55.41	21.25	23.24	48.69	46.50	44.79	41.90	38.43	49.57	30.46	32.55
	Minimum	39.53	49.44	39.25	38.59	27.26	38.04	12.40	9.42	40.14	38.12	37.80	32.30	26.99	37.10	25.31	26.75
	Maximum	60.74	62.45	57.63	61.18	64.42	67.18	30.17	32.65	55.99	52.56	54.60	54.38	43.43	57.46	32.75	39.22
	CV	0.10	0.07	0.10	0.13	0.19	0.14	0.38	0.41	0.09	0.10	0.11	0.14	0.11	0.09	0.06	0.09
	SE	1.27	1.54	1.25	1.56	1.67	1.37	3.03	3.62	0.95	1.14	1.12	1.29	0.79	0.72	0.40	0.62
GYE	Mean	1.93	2.37	1.76	2.16	1.13	1.53	0.63	0.75	2.33	2.09	1.90	1.79	1.92	2.31	1.48	1.60
	Minimum	1.04	1.24	1.28	1.28	0.59	0.76	0.32	0.33	1.73	1.30	1.15	1.04	1.23	1.24	0.97	0.98
	Maximum	2.28	5.56	2.08	2.89	1.70	2.36	1.31	1.10	3.03	2.64	2.55	2.48	2.78	3.12	1.86	2.09
	CV	0.17	0.53	0.13	0.21	0.25	0.27	0.57	0.43	0.15	0.21	0.20	0.22	0.18	0.21	0.14	0.18
	SE	0.07	0.42	0.06	0.11	0.06	0.07	0.14	0.12	0.07	0.11	0.09	0.09	0.06	0.08	0.05	0.06

SNE	Mean	35.30	34.63	34.90	40.53	32.14	32.31	28.68	32.53	48.04	44.65	42.15	42.54	49.89	46.39	48.48	49.08
	Minimum	26.42	25.06	24.44	33.14	20.50	20.35	22.48	24.26	38.65	34.06	29.59	31.51	40.46	25.01	38.44	36.53
	Maximum	42.46	40.71	47.81	48.08	46.42	42.93	44.40	34.98	60.21	53.33	50.82	55.82	64.09	61.69	57.17	63.77
	CV	0.12	0.16	0.14	0.12	0.19	0.19	0.26	0.12	0.12	0.15	0.14	0.16	0.12	0.17	0.10	0.13
	SE	0.95	2.03	1.19	1.18	1.22	1.13	2.79	1.43	1.24	1.63	1.37	1.56	1.09	1.31	1.11	1.35
TTN	Mean	18.16	16.11	18.82	15.33	16.69	15.83	26.71	23.00	15.83	15.00	19.17	14.90	16.67	13.43	19.95	18.91
	Minimum	15.00	8.00	13.00	11.00	11.00	10.00	21.00	14.00	9.00	9.00	14.00	10.00	10.00	8.00	13.00	11.00
	Maximum	22.00	19.00	25.00	20.00	25.00	22.00	33.00	30.00	22.00	24.00	30.00	24.00	26.00	25.00	39.00	28.00
	CV	0.12	0.20	0.18	0.18	0.23	0.20	0.15	0.25	0.19	0.25	0.21	0.29	0.26	0.32	0.32	0.23
	SE	0.50	1.09	0.83	0.64	0.74	0.57	1.54	2.14	0.63	0.93	0.96	0.99	0.78	0.72	1.45	0.95
TTH	Mean	14.95	13.78	18.24	13.89	18.46	17.10	18.86	16.43	15.78	15.63	19.78	16.05	17.63	14.70	20.37	20.14
	Minimum	12.00	11.00	15.00	10.00	13.00	13.00	16.00	13.00	7.00	10.00	14.00	10.00	11.00	9.00	12.00	11.00
	Maximum	18.00	18.00	24.00	17.00	26.00	22.00	24.00	23.00	21.00	24.00	29.00	28.00	27.00	30.00	30.00	30.00
	CV	0.13	0.19	0.13	0.14	0.19	0.14	0.14	0.23	0.21	0.25	0.22	0.33	0.24	0.33	0.26	0.28
	SE	0.45	0.86	0.58	0.44	0.69	0.43	0.99	1.45	0.69	0.98	1.01	1.21	0.77	0.80	1.23	1.20
DTH	Mean	90.05	89.44	94.18	90.50	96.39	96.70	101.57	99.57	98.65	97.38	100.78	99.63	105.50	104.68	110.58	105.46
	Minimum	89.00	89.00	91.00	89.00	91.00	93.00	100.00	98.00	96.00	96.00	99.00	98.00	99.00	99.00	107.00	102.00
	Maximum	92.00	91.00	96.00	92.00	105.00	102.00	103.00	101.00	101.00	100.00	105.00	102.00	116.00	112.00	114.00	109.00
	CV	0.02	0.01	0.02	0.01	0.04	0.02	0.01	0.01	0.02	0.01	0.01	0.01	0.03	0.03	0.02	0.02
	SE	0.30	0.29	0.37	0.23	0.71	0.41	0.43	0.53	0.34	0.30	0.32	0.27	0.66	0.58	0.42	0.35
TH	Mean	630.00	625.78	625.94	644.06	640.19	670.00	793.43	756.29	664.44	649.13	613.44	564.05	742.57	705.19	1087.95	1069.00
	Minimum	550.00	495.00	576.00	583.00	533.00	497.00	657.00	617.00	607.00	544.00	518.00	205.00	631.00	598.00	847.00	785.00
	Maximum	722.00	683.00	661.00	687.00	794.00	808.00	924.00	837.00	745.00	725.00	693.00	643.00	872.00	844.00	1270.00	1251.00
	CV	0.08	0.12	0.04	0.04	0.11	0.10	0.12	0.11	0.06	0.08	0.07	0.17	0.09	0.10	0.10	0.12
	SE	11.47	25.06	5.63	6.92	13.84	12.62	35.43	30.01	8.03	12.24	10.51	21.52	12.78	11.37	24.87	26.54

B	Mean	-	-	-	-	-	-	-	-	82.77	74.01	86.26	66.61	82.82	78.48	88.57	87.51
	Minimum	-	-	-	-	-	-	-	-	55.82	51.28	67.65	42.31	55.46	48.35	54.89	60.44
	Maximum	-	-	-	-	-	-	-	-	113.57	100.58	109.13	101.20	116.66	144.75	122.53	127.60
	CV	-	-	-	-	-	-	-	-	0.17	0.23	0.16	0.26	0.22	0.29	0.22	0.23
	SE	-	-	-	-	-	-	-	-	2.96	4.16	3.27	4.03	3.32	3.82	4.55	4.27
HI	Mean	-	-	-	-	-	-	-	-	0.44	0.42	0.41	0.39	0.38	0.39	0.33	0.35
	Minimum	-	-	-	-	-	-	-	-	0.36	0.34	0.34	0.26	0.31	0.21	0.28	0.27
	Maximum	-	-	-	-	-	-	-	-	0.48	0.48	0.53	0.46	0.44	0.46	0.36	0.40
	CV	-	-	-	-	-	-	-	-	0.07	0.09	0.12	0.15	0.10	0.14	0.07	0.09
	SE	-	-	-	-	-	-	-	-	0.01	0.01	0.01	0.01	0.01	0.01	0.01	0.01
GYE1	Mean	2.71	2.16	2.22	2.72	1.60	2.08	1.14	1.35	2.97	2.74	2.67	2.25	2.20	2.44	2.26	2.67
	Minimum	1.21	0.58	1.04	1.30	0.69	0.66	0.71	0.99	1.90	0.49	0.82	0.45	0.31	0.03	0.79	1.71
	Maximum	3.83	3.50	3.35	3.86	2.43	3.77	1.38	1.68	3.68	3.35	3.57	3.28	3.49	4.35	3.24	3.39
	CV	0.22	0.41	0.28	0.30	0.34	0.40	0.22	0.17	0.14	0.21	0.20	0.30	0.39	0.50	0.20	0.17
	SE	0.13	0.28	0.14	0.17	0.10	0.15	0.09	0.09	0.08	0.11	0.10	0.13	0.13	0.15	0.09	0.08
SNE1	Mean	45.83	36.90	38.90	47.13	40.50	43.30	38.14	42.29	58.37	55.04	53.97	49.64	55.26	45.74	67.93	71.60
	Minimum	19.00	15.00	19.00	24.00	19.00	13.00	25.00	31.00	44.00	43.00	33.00	23.00	11.00	2.00	56.00	55.00
	Maximum	60.00	58.00	58.00	64.00	58.00	67.00	46.00	50.00	76.00	67.00	69.00	64.00	77.00	93.00	94.00	91.00
	CV	0.22	0.37	0.29	0.26	0.28	0.31	0.23	0.16	0.12	0.11	0.14	0.19	0.31	0.50	0.11	0.12
	SE	2.12	4.33	2.56	2.55	2.14	2.44	3.26	2.49	1.29	1.13	1.43	1.82	2.46	2.75	1.39	1.55
EL1	Mean	6.22	5.79	5.89	5.86	6.45	7.13	8.89	8.94	6.84	6.57	6.39	6.38	7.68	7.93	11.45	12.05
	Minimum	5.50	5.60	5.20	5.30	5.10	5.90	8.20	8.20	5.90	5.90	5.80	5.70	6.30	6.60	10.50	11.20
	Maximum	6.70	6.30	6.30	6.60	7.80	8.60	9.50	9.60	7.80	7.20	6.90	7.20	11.60	10.20	12.60	13.50
	CV	0.06	0.05	0.06	0.06	0.10	0.09	0.05	0.05	0.06	0.04	0.05	0.06	0.12	0.09	0.04	0.05
	SE	0.07	0.08	0.08	0.07	0.12	0.12	0.18	0.18	0.08	0.05	0.06	0.07	0.13	0.09	0.09	0.11

SPNE1	Mean	14.65	13.40	14.25	14.57	14.36	15.97	15.29	16.14	14.43	13.50	13.66	13.14	15.55	16.71	19.72	20.00
	Minimum	13.00	12.00	13.00	13.00	12.00	13.00	14.00	14.00	13.00	12.00	12.00	12.00	12.00	13.00	17.00	18.00
	Maximum	17.00	15.00	17.00	16.00	17.00	19.00	16.00	18.00	17.00	16.00	18.00	15.00	20.00	22.00	22.00	22.00
	CV	0.06	0.06	0.07	0.05	0.07	0.09	0.05	0.08	0.07	0.08	0.10	0.07	0.12	0.14	0.06	0.06
	SE	0.18	0.27	0.23	0.14	0.20	0.27	0.29	0.46	0.18	0.21	0.25	0.18	0.27	0.27	0.20	0.21
SPS1	Mean	3.13	2.73	2.71	3.23	2.82	2.71	2.50	2.63	4.04	4.09	3.96	3.78	3.61	2.79	3.45	3.59
	Minimum	1.36	1.15	1.46	1.60	1.36	0.87	1.56	1.82	3.14	2.87	2.54	1.60	0.73	0.12	2.95	2.75
	Maximum	4.29	4.00	3.71	4.21	4.00	3.94	3.07	3.13	5.07	4.83	4.77	4.75	5.14	5.17	4.48	4.72
	CV	0.22	0.34	0.26	0.25	0.27	0.29	0.22	0.15	0.09	0.11	0.12	0.18	0.32	0.50	0.11	0.13
	SE	0.14	0.29	0.16	0.17	0.14	0.14	0.21	0.15	0.07	0.08	0.09	0.13	0.17	0.17	0.07	0.08
FLW	Mean	1.81	1.71	2.01	1.79	1.80	2.06	1.64	1.71	1.98	2.01	2.01	1.83	1.85	2.01	1.83	2.03
	Minimum	1.60	1.60	1.70	1.60	1.40	1.60	1.60	1.50	1.70	1.80	1.60	1.30	1.50	1.70	1.20	1.80
	Maximum	2.00	1.90	2.30	2.00	2.30	2.50	1.70	1.90	2.30	2.20	2.50	2.10	2.20	2.50	2.20	2.30
	CV	0.06	0.06	0.08	0.07	0.12	0.11	0.03	0.08	0.08	0.07	0.10	0.09	0.09	0.08	0.13	0.06
	SE	0.02	0.04	0.04	0.03	0.04	0.04	0.02	0.05	0.03	0.03	0.04	0.03	0.02	0.02	0.04	0.02
FLL	Mean	28.80	29.46	26.45	26.60	26.85	29.75	24.23	26.49	28.21	28.89	28.95	28.13	26.90	30.16	22.82	27.17
	Minimum	22.90	24.10	23.40	24.30	20.40	21.80	21.50	20.70	21.20	25.20	21.90	22.10	20.00	17.70	13.80	19.50
	Maximum	33.50	33.00	32.40	30.60	31.40	38.60	28.40	33.70	32.40	32.00	34.20	31.40	34.40	40.70	29.30	35.30
	CV	0.10	0.09	0.09	0.07	0.11	0.13	0.10	0.19	0.11	0.06	0.11	0.09	0.14	0.14	0.16	0.16
	SE	0.62	0.84	0.51	0.37	0.54	0.69	0.88	1.93	0.57	0.31	0.58	0.48	0.54	0.52	0.69	0.79
FLA	Mean	39.33	37.88	39.92	35.82	36.41	46.39	29.865	34.43	42.17	43.54	43.89	38.77	37.38	45.62	31.83	41.56
	Minimum	27.48	28.92	30.22	30.12	22.26	29.43	25.8	23.287	28.62	36.86	26.28	25.64	24.75	23.90	15.39	27.79
	Maximum	48.15	47.03	49.83	45.90	50.54	66.59	34.08	45.495	55.55	52.80	64.13	47.25	51.98	75.19	45.99	60.89
	CV	0.15	0.13	0.15	0.12	0.19	0.21	0.105	0.26	0.17	0.10	0.18	0.15	0.19	0.18	0.26	0.20
	SE	1.21	1.60	1.32	0.90	1.32	1.76	1.181	3.388	1.28	0.83	1.45	1.07	1.04	1.00	1.55	1.50

FL-1W	Mean	1.47	1.46	1.51	1.49	1.39	1.60	1.43	1.40	1.59	1.49	1.56	1.46	1.48	1.59	1.64	1.72
	Minimum	1.30	1.30	1.40	1.20	1.00	1.20	1.30	1.20	1.40	1.20	1.20	1.20	1.10	1.10	1.50	1.50
	Maximum	1.60	1.60	1.70	1.60	1.70	2.20	1.50	1.60	1.80	1.70	1.90	1.70	1.90	2.00	1.90	2.00
	CV	0.07	0.07	0.07	0.07	0.13	0.13	0.05	0.09	0.08	0.09	0.11	0.10	0.12	0.12	0.06	0.08
	SE	0.02	0.03	0.02	0.02	0.04	0.04	0.03	0.05	0.02	0.03	0.03	0.03	0.03	0.02	0.02	0.02
FL-1L	Mean	29.52	29.49	30.16	30.57	30.29	33.77	27.99	30.13	30.07	29.48	29.36	27.92	30.23	32.19	26.89	32.25
	Minimum	25.90	23.30	25.80	27.60	23.50	26.60	22.60	21.80	21.10	26.50	20.50	23.10	19.90	23.70	23.70	23.30
	Maximum	33.90	31.50	33.90	33.20	36.50	41.60	34.60	33.70	35.50	31.70	35.60	32.20	35.70	39.60	30.70	36.70
	CV	0.07	0.08	0.07	0.05	0.10	0.10	0.14	0.15	0.10	0.05	0.10	0.09	0.10	0.10	0.06	0.09
	SE	0.42	0.78	0.47	0.32	0.60	0.59	1.46	1.71	0.58	0.26	0.53	0.51	0.44	0.37	0.32	0.53
FL-1A	Mean	32.61	32.43	34.20	34.25	31.77	40.87	29.971	31.771	35.90	33.07	34.53	30.68	33.84	38.55	33.12	41.58
	Minimum	26.13	22.72	28.04	24.84	17.63	25.65	25.425	19.62	23.74	25.84	22.59	22.52	16.42	23.11	27.23	27.96
	Maximum	40.68	37.68	40.68	39.84	42.48	59.07	36.33	38.52	47.93	40.42	50.73	38.12	48.20	55.01	40.33	50.30
	CV	0.12	0.14	0.11	0.10	0.20	0.19	0.145	0.191	0.14	0.12	0.17	0.17	0.19	0.18	0.11	0.11
	SE	0.81	1.47	0.86	0.74	1.20	1.43	1.645	2.29	0.97	0.78	1.12	1.08	0.92	0.84	0.68	0.84

[1] Whole plant traits - GY: grain yield per plant (g), SN: seed number per plant, TKW: thousand kernel weight (g), GYE: grain yield per ear (g), SNE: seed number per ear, TTN: total productive tiller number, TTH: tiller number at heading, DTH: days to heading, TH: plant height (mm), B: biomass per plant (g), HI: harvest index; Main shoot traits - GYE1: grain yield per ear (g), SNE1: seed number per ear, SPNE1: spikelet number per ear, EL1: ear length (cm), SPS1: seeds per spikelet, FLW: flag leaf width (cm), FLL: flag leaf length (cm), FLA: flag leaf area (cm2), FL-1W: flag leaf-1 width (cm), FL-1L: flag leaf-1 length (cm), FL-1A: flag leaf-1 area (cm^2)

[2] CV = coefficient of variation

[3] SE = standard error of arithmetic mean

Supplementary Table S2. Two-year means and standard errors (SE) as from ANOVA-GLM analyses of yield-contributing traits of the durum and bread wheat 7AgL recombinant lines and their respective controls.

Trait[1]	R5-2-10 HOM+		R5-2-10 HOM-		R112-4 HOM+		R112-4 HOM-		R23-1 HOM+		R23-1 HOM-		T4		Thatcher	
	Mean	SE	Mean	SE	Mean	SE	Mean	SE	Mean	SE	Mean	SE	Mean	SE	Mean	SE
GY	35.72	1.20	32.73	1.61	34.13	1.31	29.67	1.27	25.13	1.04	26.93	0.97	21.98	1.44	23.21	1.41
SN	695.16	23.89	622.21	35.19	721.50	25.80	623.03	25.06	677.94	20.54	555.82	19.12	842.26	48.06	834.71	47.16
TKW	51.67	0.88	51.45	1.30	47.77	0.95	47.63	0.93	41.77	0.76	52.26	0.71	25.33	0.62	27.37	0.61
GYE	2.14	0.07	2.23	0.09	1.84	0.07	1.98	0.07	1.51	0.06	1.90	0.05	1.04	0.06	1.17	0.06
SNE	41.73	0.95	39.66	1.40	38.56	1.03	41.63	1.00	40.71	0.82	39.29	0.76	38.65	1.27	41.07	1.26
TTN	16.94	0.57	15.46	0.77	18.89	0.62	15.13	0.60	16.76	0.49	14.56	0.46	23.03	1.16	20.68	1.15
TTH	15.28	0.57	14.57	0.77	18.92	0.63	14.98	0.61	18.07	0.50	15.85	0.46	19.52	1.08	18.15	1.06
DTH	94.35	0.38	93.47	0.52	97.46	0.42	95.08	0.41	101.02	0.33	100.73	0.31	106.10	0.33	102.61	0.33
TH	647.95	9.50	638.17	12.80	617.59	10.37	605.74	10.22	691.70	8.26	685.00	7.69	939.89	24.86	916.72	24.39
GYE1	2.84	0.11	2.45	0.15	2.45	0.12	2.48	0.12	1.90	0.10	2.27	0.09	1.69	0.10	2.02	0.10
SNE1	52.13	2.02	45.96	2.69	46.56	2.12	48.36	2.05	47.90	1.74	44.57	1.62	52.80	1.75	57.12	1.74
EL1	6.53	0.08	6.17	0.10	6.14	0.08	6.11	0.08	7.07	0.07	7.51	0.06	10.16	0.11	10.50	0.11
SPNE1	14.53	0.18	13.49	0.24	13.97	0.19	13.87	0.18	14.98	0.15	16.36	0.14	17.53	0.20	18.12	0.20
SPS1	3.59	0.13	3.40	0.17	3.34	0.13	3.50	0.13	3.21	0.11	2.74	0.10	2.95	0.09	3.11	0.09
FLW	1.90	0.02	1.87	0.03	2.01	0.02	1.81	0.02	1.82	0.02	2.05	0.02	1.74	0.04	1.88	0.04
FLL	28.50	0.46	29.20	0.61	27.70	0.48	27.36	0.46	26.89	0.39	30.00	0.37	23.75	0.81	27.21	0.81
FLA	40.70	0.98	40.87	1.30	41.97	1.02	37.31	0.99	36.99	0.84	46.22	0.78	31.16	1.65	38.63	1.64
FL-1W	1.53	0.02	1.49	0.03	1.54	0.02	1.48	0.02	1.44	0.02	1.61	0.02	1.53	0.02	1.56	0.02
FL-1L	29.80	0.37	29.46	0.49	29.76	0.38	29.23	0.39	30.26	0.32	32.93	0.29	27.58	0.59	31.33	0.58
FL-1A	34.16	0.77	32.98	1.00	34.48	0.78	32.46	0.80	32.85	0.65	39.87	0.60	31.66	0.91	36.92	0.90

[1] Whole plant traits - GY: grain yield per plant, SN: seed number per plant, TTN: total productive tiller number, TTH: tiller number at heading, TH: plant height, B: biomass per plant; Main shoot traits - GYE1: grain yield per ear, SNE1: seed number per ear, SPNE: spikelet number per ear, EL: ear length, FLW: flag leaf width, FLL: flag leaf length, FL-1W: flag leaf-1 width, FL-1L: flag leaf-1 length.

Supplementary Table S3. Classification matrix (cases in row categories classified into columns) of discriminant analysis (DA) applied to HOM+ durum wheat NIRLs and to their HOM- controls for traits recorded in A) 2009 and 2010, B) 2010 only.

A)	R112-4 HOM+	R112-4 HOM-	R23-1 HOM+	R23-1 HOM-	R5-2-10 HOM+	R5-2-10 HOM-	Total
R112-4 HOM+	**26**	3	2	2	1	3	37
R112-4 HOM-	4	**23**	2	1	7	3	40
R23-1 HOM+		1	**49**	4	4		58
R23-1 HOM-			1	**50**	3		54
R5-2-10 HOM+	3	6		4	**22**	4	39
R5-2-10 HOM-	2	4	2	5	4	**13**	30
%correct	74	62	88	76	54	57	**71**
B)							
R112-4 HOM+	**10**	2			2	2	16
R112-4 HOM-	3	**15**		1		1	20
R23-1 HOM+			**30**	1	2		33
R23-1 HOM-				**26**	2		28
R5-2-10 HOM+	3			2	**13**	2	20
R5-2-10 HOM-	2	1		3	3	**9**	18
% correct	56	83	100	79	59	64	**76**

Supplementary Table S4. Eigenvalues, total variance and cumulative variance of discriminant analysis (DA) for traits of HOM+ and HOM- plants of durum wheat NIRLs for A) 2009 and 2010, B) 2010 only; DF = discriminant function.

A)	DF1	DF2	DF3	DF4	DF5
Eigenvalues	1.658	1.078	0.322	0.12	0.062
% total variance	52.2	32.3	9.9	3.7	1.9
% cumulative variance	52.2	84.5	94.4	98.1	100
B)					
Eigenvalues	2.246	1.685	0.553	0.14	0.08
% total variance	47.8	35.8	11.7	3.0	1.7
% cumulative variance	47.8	83.6	95.3	98.3	100

Supplementary Table S5. Canonical discriminant functions (DFs) standardized by within variances of discriminant analysis (DA) for traits of HOM+ and HOM- plants of durum wheat NIRLs for A) 2009 and 2010, B) 2010 only. Values in bold indicate traits mainly contributing to the classification at a specific DF.

Trait[1]	DF1	DF2	DF3	DF4	DF5
A)					
FLW	0.260	0.336	**1.158**	0.004	0.294
FLL	0.147	0.165	-0.339	0.044	0.345
FL_1W	0.339	-0.145	-0.272	-0.087	-0.182
FL_1L	-0.445	0.112	-0.295	-0.212	-0.126
EL1	-0.420	0.620	-0.036	0.258	-0.103
TH	-0.421	-0.066	0.291	0.298	0.301
SNE1	-0.457	-0.786	0.116	**1.979**	0.491
SPNE1	-0.359	0.195	-0.131	-0.213	-0.550
GYE1	**0.826**	0.515	-0.569	**-1.758**	-0.625
TTH	-0.157	0.175	0.359	-0.590	0.380
TTN	0.164	-0.113	0.352	0.781	-0.628
GY	**0.727**	**1.309**	0.428	1.738	0.428
SN	-0.547	**-1.502**	-0.455	-1.702	-0.677
B)					
FLW	0.068	0.285	0.775	**0.850**	0.634
FLL	0.341	0.011	-0.487	0.116	-0.045
FL_1W	0.604	-0.472	-0.102	-0.520	-0.591
FL_1L	-0.440	0.137	0.010	-0.229	-0.122
EL1	-0.563	0.231	0.065	-0.298	0.046
TH	-0.505	-0.169	0.464	0.130	0.324
SNE1	-0.247	-0.652	0.733	-0.249	0.286
SPNE1	-0.268	0.305	-0.555	0.609	-0.541
GYE1	0.234	0.668	-0.405	-0.130	-0.287
TTH	0.376	0.127	-0.479	0.564	0.545
TTN	0.233	0.181	**0.934**	0.290	-0.663
GY	0.156	**1.512**	**1.574**	-0.636	0.171
SN	0.055	**-1.785**	-0.925	0.131	-0.494
B	**-0.732**	-0.026	-0.863	0.103	-0.023

[1] Whole plant traits - GY: grain yield per plant, SN: seed number per plant, TTN: total number of ear-bearing tillers, TTH: tiller number at heading, TH: plant height, B: biomass per plant; Main shoot traits - GYE1: grain yield per ear, SNE1: seed number per ear, SPNE1: spikelet number per ear, EL1: ear length, FLW: flag leaf width, FLL: flag leaf length, FL-1W: flag leaf-1 width, FL-1L: flag leaf-1 length

Supplementary Table S6. Mean squares from ANOVA-GLM for yield and yield-related traits of the bread wheat T4 NIL and its control cv. Thatcher determined in 2009 and/or 2010. (A = whole plant traits in 2009+2010; B = whole plant traits in 2010; C = main shoot traits in 2009 + 2010); GY: grain yield per plant (g), SN: seed number per plant, TTN: total productive tiller number, TTH: tiller number per plant at heading, TKW: thousand-kernel weight (g), GYE: grain yield per ear (g), SNE: seed number per ear, TH: plant height (mm), DTH: days to heading; FLW: flag leaf width (cm), FLL: flag leaf length (cm), FLA: flag leaf area (cm2), FL-1W: flag leaf-1 width (cm), FL-1L: flag leaf-1 length (cm), FL-1A: flag leaf-1 area (cm2), EL1: ear length (cm), SNE1: seed number per main shoot ear, SPNE1: spikelet number per main shoot ear, SPS1: seeds per spikelet, GYE1: grain yield per main shoot ear (g), B: dry biomass of the whole above-ground part of the plant (g), HI: harvest index; *, **, *** indicate significant F values at *P*<0.05, 0.01, and 0.001, respectively.

A)

Factor	df	GY	SN	TTN	TTH	TKW	GYE	SNE	TH	DTH
Y	1	1888.0 ***	408607.9 **	269.8 **	63.9	1057.5 ***	7.9 ***	3397.1 ***	910485.0 ***	521.0 ***
T	1	623.6 ***	54450.5	47.8	5.1	565.9 ***	0.4 *	4.7	6691.5	1.0
Y x T	1	276.5 *	12626.0	25.2	65.8	646.0 ***	0.9 **	36.3	14650.1	0.4
7AgL(G)	1	11.4	1142.1	63.5	22.8	40.3 *	0.2	66.9	5032.5	126.7 ***
R(Y)	4	24.8	30489.6	16.1	38.2	2.0	0.0	26.6	7933.3	8.7 **
Y x 7AgL(G)	1	0.5	918.4	21.7	14.1	0.3	0.0	35.5	587.2	23.6 **
T x 7AgL(G)	1	14.6	46933.4	0.4	0.6	2.6	0.0	47.6	16533.7	1.4
T x Y x 7AgL(G)	1	38.7	12483.6	36.7	20.6	4.1	0.0	35.3	1348.7	1.4
Error	43	41.2	46839.8	26.6	23.3	7.8	0.1	32.2	12578.0	2.2
R^2		0.60	0.27	0.36	0.29	0.84	0.76	0.73	0.66	0.90

B)

Factor	df	B	HI
7AgL(G)	1	9.2	0.004 *
T	1	396.9	0.000
R	2	652.0	0.003 *
T x 7AgL(G)	1	724.3	0.000
Error	35	368.7	0.001
R^2		0.17	0.31

C)

Factor	df	FLW	FLL	FLA	FL-1W	FL-1L	FL-1A	EL1	SNE1	SPNE1	SPS1	GYE1
Y	1	0.65 ***	9.6	144.2	0.75 ***	0.6	407.4 ***	87.9 ***	9533.8 ***	179.2 ***	10.4 ***	16.3 ***
T	1	0.03	25.0	75.7	0.03	64.7 **	161.4 **	0.1	55.3	10.7 **	0.0	0.1
Y x T	1	0.00	79.0 *	163.8	0.00	45.6 *	30.1	0.0	3.4	0.6	0.0	0.0
7AgL(G)	1	0.22 **	133.0 **	620.8 **	0.01	156.2 ***	306.9 ***	1.3 *	207.3	3.8 *	0.3	1.2 *
R(Y)	4	0.05	29.5	127.1	0.00	2.6	4.0	0.3	12.0	0.8	0.1	0.0
T x 7AgL(G)	1	0.00	9.6	25.0	0.04	0.0	20.9	0.2	99.0	0.4	0.2	0.1
Y x 7AgL(G)	1	0.04	8.3	54.8	0.03	28.4	113.4 *	0.8	4.6	0.9	0.0	0.1
T x Y x 7AgL(G)	1	0.06	23.1	121.5	0.01	0.0	2.4	0.0	82.6	0.0	0.4	0.2
Error	61	0.03	14.3	59.5	0.01	7.5	18.1	0.3	67.2	0.9	0.2	0.2
R^2		0.49	0.38	0.41	0.58	0.53	0.62	0.85	0.72	0.80	0.50	0.62

Supplementary Table S7. ANOVA-GLM summary table for endogenous ABA content in juvenile spikes of A) durum wheat NIRLs and B) bread wheat T4 NIL, and for corresponding relative water content (RWC) of C) durum wheat NIRLs and D) bread wheat T4 NIL; SS = sum of squares, MS = mean squares.

A)

Factor	df	Type III SS	MS	F-ratio	p-value
G	2	1157.07	578.54	0.864	0.428
7AgL(G)	3	82.24	27.41	0.041	0.989
Error	47	31480.07	669.79		

B)

Factor	df	Type III SS	MS	F-ratio	p-value
7AgL(G)	1	10.25	10.25	0.009	0.927
Error	10	11552.43	1155.24		

C)

Factor	df	Type III SS	MS	F-ratio	p-value
G	2	0.011	0.005	6.389	0.004
7AgL(G)	3	0.004	0.001	1.593	0.204
Error	47	0.039	0.001		

D)

Factor	df	Type III SS	MS	F-ratio	p-value
7AgL(G)	1	0.001	0.001	0.948	0.353
Error	10	0.008	0.001		
